# Evaluation of risk strategies for supply chain sustainability with interval-valued neutrosophic fuzzy EDAS

**DOI:** 10.1016/j.heliyon.2024.e38607

**Published:** 2024-09-27

**Authors:** Ecenur Alioğulları, Yusuf Sait Türkan, Emre Çakmak, Erfan Babaee Tirkolaee

**Affiliations:** a Faculty of Engineering, Department of Industrial Engineering, Istanbul University, Cerrahpasa, Istanbul, Turkey; b Department of Industrial Engineering, Istinye University, Istanbul, Turkey; c Department of Industrial Engineering and Management, Yuan Ze University, Taoyuan, Taiwan; d Department of Mechanics and Mathematics, Western Caspian University, Baku, Azerbaijan

**Keywords:** Interval-valued neutrosophic fuzzy EDAS, Multi-criteria decision-making, Risk strategy, Supply chain sustainability, Automotive industry

## Abstract

Sustainability means the most effective and efficient use of existing resources to meet the needs of future and current generations. Nowadays, with the increase in global environmental problems as well as social and economic problems, sustainability in the supply chain of the automotive industry has become increasingly important. In our study, after conducting an extensive literature review in the automotive industry, consulting with experts, and identifying 35 different Sustainability Risks across 5 sustainability dimensions, we proposed a total of 40 risk strategies to mitigate these risks. Using the evaluations of automotive supply chain experts from one of Turkey's leading logistics companies, risk strategies were ranked using the Interval-Valued Neutrosophic Fuzzy Evaluation based on Distance from Average Solution (IVN Fuzzy EDAS) method, and the best strategies were determined. Accordingly, it has been determined that the optimal approach for companies is to establish and implement legal compliance procedures and programs while adhering to prevailing regulations and policies. It aims to make a useful contribution to the literature in terms of covering this subject for the first time with fuzzy EDAS method, adding new sustainability risk dimensions, and developing broad-level risk and risk prevention strategies on the subject. In this study, the interval-valued neutrosophic fuzzy EDAS method was applied for the first time to the issue of sustainability risks in the automotive industry. At the same time, this study aims to contribute to the literature by adding new dimensions to sustainability.

## 1 Introduction

In recent years, the automotive industry has been undergoing major changes. With the emergence of electric vehicles, both internal combustion and electric powertrains share the same space in vehicle production lines. In response to customer demands and expectations, vehicles now incorporate more parts than ever before. A component manufacturer needs to supply a larger quantity of components than Original Equipment Manufacturers (OEMs). Production centers for semi-finished goods have relocated to regional geographies. Zero-emission technologies are developing rapidly, necessitating prompt and efficient adaptations. The implementation of these new technologies brings with it novel production and supply complications. The expansion of the supply chain in the automotive industry has brought with it social and economic risks, especially related to the environment. In this context, new risks related to sustainability in the automotive industry are emerging, and it is of great importance to develop strategies to address these risks in addition to identifying them.

Organizations aim to increase their profitability in the long and short term, enhance their current business performance, and distribute sustainable products and services to future generations [1]. Sustainable Supply Chain Management (SSCM) is of great importance in realizing this goal. The goal of SSCM is to optimize activities and minimize resource usage to maximize business profits while maintaining environmental, social, and economic sustainability. Additionally, the aim is to minimize waste production within the enterprise [2]. SSCM involves the efficient and effective management of information and capital flows, as well as the creation of supply chains that coordinate social, economic, and environmental factors, which are the three dimensions of sustainability. Conceptual models of Supply Chain Risk Management (SCRM) have been created through a number of studies, such as Rao and Goldsby [3], Foerstl et al. [4], Ritchie and Brindley [5]. While terminologies may vary, a standard SCRM procedure involves four steps: identifying risks, evaluating risks based on probability and impact, mitigating risks, and monitoring risks [6,7]. Researchers are now focusing on the potential risks involved in achieving sustainability objectives and the necessary research to mitigate these risks. Several recent studies have investigated Sustainability Risks (SRs) in the supply chain. In the textile industry, Raian et al. [8] employed the fuzzy synthetic evaluation method. Bathrinath et al. [9] focused on the sugar production industry and explored Delphi, Analytical Hierarchy Process (AHP), and Best-Worst Method (BMW) methods. In freight transportation systems, Shankar et al. [10] statistically modeled SRs using the D-number concept and heuristic fuzzy sets. Chowdhury et al. [11] utilized dynamic capability to enhance supply chain sustainability within the ready-to-wear apparel sector. Awasthi et al. [12] proposed an integrated model based on the AHP-Vise Kriterijumska Optimizacija I Kompromisno Resenje (VIKOR) approach for sustainable global supplier selection and reducing existing risks. Classical Multi-Criteria Decision-Making (MCDM) models, such as AHP, BWM, and VIKOR, have found extensive use in the context of supply chain sustainability. Although these models provide valuable insights, they frequently fail to adequately address the inherent uncertainty and ambiguity associated with SRs, particularly in the dynamic and complex automotive industry. The objective of this study is to identify the key SR factors in the automotive sector and to determine the relative importance of the corresponding risk strategies. This research represents a pioneering effort in the automotive sector in Turkey, as it is the first time that the Interval-Valued Neutrosophic (IVN) Fuzzy Evaluation based on Distance from Average Solution (EDAS) method has been applied in this field. It introduces new dimensions of sustainability and addresses SRs in a comprehensive manner, thereby making a notable contribution to the existing literature on the subject. Following an extensive review of the literature and consultations with experts, 35 SRs were identified and 40 risk prevention strategies were proposed. These were validated by four experts from a leading logistics company in Turkey. This study employs the IVN fuzzy EDAS method, which incorporates neutrosophic sets to effectively manage the ambiguity and uncertainty of risk factors. This methodological innovation provides a more accurate risk estimation tool for Decision-Makers (DMs) in the automotive industry, addressing gaps in classical MCDM models. The consistency and robustness of the method were verified through a sensitivity analysis, demonstrating its effectiveness in handling SRs. In addition to defining SRs in the automotive sector, this study also considers managerial and organizational dimensions in conjunction with social, economic, and environmental dimensions. This study makes a significant contribution to the field of supply chain sustainability in the automotive industry through its comprehensive risk identification and the use of the IVN fuzzy EDAS approach to prioritize risk prevention strategies. The remainder of this paper is organized as follows: Section 2 presents the background of the study, including a comprehensive review of the literature on sustainability in the automotive industry. Section 3 outlines the methodology, providing a detailed account of the scope and execution steps. Section 4 presents the case study, findings, and sensitivity analysis. In conclusion, Section 5 presents the key insights and outlines the future research directions.

## 2 Background

SRs in the supply chain are defined as social, environmental, and economic threats that have the potential to impede an organization's long-term functionality and sustainability. These risks are particularly pronounced in dynamic and complex sectors like the automotive industry, where supply chains are extensive and intricate. Prioritizing risk strategies related to these SRs is crucial because it allows organizations to allocate resources effectively, mitigate potential disruptions, and ensure a balanced approach to economic, social, and environmental sustainability. By prioritizing these strategies, organizations can not only safeguard their operations but also enhance their resilience and adaptability in the face of emerging challenges. As defined by Amabile [13], the term “sustainability” encompasses the social, environmental, and economic prerequisites necessary to maintain the continued functionality of an entity over an extended period of time. The concept of sustainability can be divided into three primary categories. The first of these is economic sustainability, which refers to a business's capacity to generate profits while maintaining a robust financial framework. The term “environmental sustainability” is defined as the utilization of Earth's resources in a manner that does not endanger the environment. Concurrently, measures are implemented to prevent the detrimental impact of resources on the environment. The primary objective of social sustainability is to enhance the working and living conditions of staff members, clients, society, and future generations [14]. This study made a significant contribution to the existing literature by incorporating organizational and managerial considerations into the identification of SRs. The organizational dimension of the study encompasses the sub-dimensions that are essential for the effective planning and implementation of supply chain processes. The managerial dimension encompasses the approval of top management and the obligations that are requisite for the assurance of the sustainability of supply chain operations.

A number of studies have addressed various aspects of supply chain sustainability and the potential risks associated with it. In a recent study, Ahmed et al. [15] employed an integrated approach integrating the worst-case method with the Bayesian method to identify critical technologies for environmentally sustainable supply chains in emerging economies. In a review of 78 papers, Burgess et al. [16] addressed supply chain quality management practices in sustainable food networks. Riaz et al. [17] introduced a novel approach to multi-stage decision-making problems in effective urban supply chain management, namely the use of dynamic global fuzzy aggregation operators. Manurung et al. [18] put forth a conceptual framework of supply chain resilience towards sustainability from a service-dominant logic perspective. Other similar noteworthy studies can be found in Refs. [[19], [20], [21], [22], [23]]. Seuring et al. [24] underscored the frequently neglected social dimension in the field of supply chain sustainability research.

Han and Um [25] examined the potential SRs present within the supply chain. They proposed four fundamental risk mitigation strategies with the aim of enhancing resilience in the context of global uncertainty. To investigate these strategies, they employed structural equation modelling. Göçer et al. [26] conducted a study investigating the implementation of SR mitigation strategies within the food supply chain in an emerging economy. The study employed focus group interviews with 29 companies and 9 senior managers. Valizadeh et al. [27] addressed the issue of SRs in closed-loop supply chains using a hybrid mathematical model and a questionnaire comprising four SRs and 18 sub-criteria. Vidal et al. [28] enhanced the SR methodologies employed by a Peruvian paint company through the utilization of fuzzy Decision Making Trial and Evaluation Laboratory (DEMATEL) and fuzzy AHP techniques. Attia and Uddin [29] employed a hybrid evaluation approach utilizing the fuzzy synthetic evaluation methodology to survey 32 factors pertaining to the flexible and sustainable supply chain. Kähkönen et al. [30] developed strategies for managing sustainability-related risks faced by companies through a multiple case study of 25 companies forming five multi-tier supply chains. Aman et al. [31] conducted an analysis of 164 studies on the management of SRs among the Base of Pyramid (BoP) in order to guarantee that companies are able to achieve their performance targets. Sawik [32] put forth a multi-criteria optimization approach to address SRs in space exploration, taking into account environmental sustainability, resource utilization, ethical concerns, international cooperation, and long-term planning. Sutrisno and Kumar [33] presented an analysis of additional failure modes, Failure Mode and Effects Analysis (FMEA), and Preference Selection Index (PSI) methods for the assessment of supply chain SRs in the context of a Small and Medium-sized Enterprise (SME) in a developing country. Vieira et al. [34] presented a case study based on a simulation-based decision support system and multi-objective optimization in the context of supply chain SR. Jianying et al. [35] identified managerial risks as arising from decision-making errors by inexperienced and unskilled management, which can significantly impede supply chain coordination. Alimohammadlou and Khoshsepehr [36] underscored the necessity of incorporating economic, social, and environmental considerations into the framework of sustainable development, particularly within the context of operational business processes. Zhang and Song [21] highlifhted the significance of operational complexity, trust risk, regulatory uncertainty, and the absence of standards as sub-dimensions of social SRs. Song et al. [37] classified the identified risk factors into four main categories: operational, environmental, economic, and social. Klumpp and Zijm [38] underscored the significance of the managerial approach in mitigating social SRs in the supply chain. Bansal and Roth [39] similarly emphasized the importance of limited financial resources and managers' attitudes within the sustainability sub-dimensions. A synthesis of these studies reveals that organizations must prioritize the social dimension. As concerns about social aspects have grown, the sub-dimensions assessed under this category have broadened. Concurrently, the domains of management and organization have acquired considerable prominence in the context of sustainable supply chain practices, a shift from their previous marginalization within the social dimension. These areas have now been established as discrete categories.

A variety of decision analysis methods have been utilized to assess sustainability in the automotive industry. Salvado et al. [40] proposed a social and environmental sustainability index using the AHP method, which provides firms with insights into their economic positions and performance at both the individual and supply chain levels. Lenort et al. [41] investigated the factors the factors influencing the automotive industry's prioritization of sustainable development goals, employing frequency analysis and the Promethee method. Yousefi and Tosarkani [42] developed a comprehensive methodology for the management of logistics process risks in automotive supply chains, identifying critical risks and proposing effective mitigation strategies. Notwithstanding the aforementioned insights, previous studies frequently neglect to address the intrinsic uncertainty and ambiguity associated with SRs, particularly within the intricate automotive industry. This gap in the literature highlights the need for more robust methodologies. The EDAS method was selected for its recent development, effectiveness in solving conflicting problems, and superior results in risk-related issues [[43], [44], [45], [46], [47], [48], [49]].

This study represents a significant advancement by introducing the IVN fuzzy EDAS method to SRs in the automotive supply chain. Unlike classical MCDM models, the IVN fuzzy EDAS method effectively handles the ambiguity and uncertainty of risk factors, providing a more accurate risk estimation tool. This methodological innovation addresses a critical gap in the existing literature by offering a nuanced approach that accommodates the industry's complexity. Moreover, the study integrates organizational and managerial dimensions into the sustainability framework, an aspect often overlooked in previous research. By doing so, it offers a holistic understanding of SRs, encompassing social, economic, and environmental dimensions, and the roles of organizational structure and managerial oversight. Practical validation through expert consultations from a leading logistics company in Turkey enhances the findings' reliability and applicability. The sensitivity analysis conducted demonstrates the robustness and consistency of the IVN fuzzy EDAS method, solidifying its relevance and utility. In conclusion, this study fills a significant gap in the literature by applying a novel methodological approach to SR management in the automotive sector, providing valuable insights and practical tools for DMs aiming to enhance sustainability and resilience in their supply chains.

## 3 Methodology

**Fig. 1:**
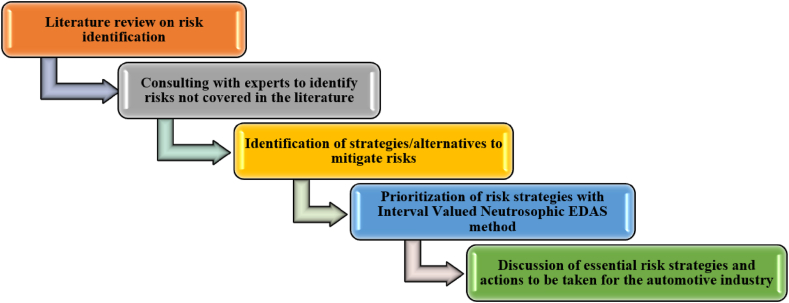
Stages of the suggested methodology.

A comprehensive five-step methodology was employed to determine SRs specific to the automotive industry, as illustrated in Fig. 1. The initial phase of the study entailed a comprehensive examination of existing literature, with a particular emphasis on the five core dimensions of sustainability: social, environmental, economic, organizational, and managerial. The objective of this review was to identify pertinent risks within the automotive sector. In the second phase, the perspectives of domain experts were consulted with the objective of enhancing the comprehension of SRs. This entailed conducting face-to-face interviews with experts, which facilitated the identification of supplementary SRs that were not previously addressed in the existing literature. These expert consultations proved invaluable in terms of providing insights and ensuring that the identified risks were comprehensive and current.

In the third phase, strategies for mitigating the identified risks were developed. The strategies were formulated based on the insights gained from the literature review and expert interviews, ensuring their practicality and applicability to the automotive industry. The fourth step entailed the application of the IVN Fuzzy EDAS technique. This sophisticated methodology was employed to prioritize the risk mitigation strategies, thereby facilitating the identification of a core set of actions to address the identified SRs. The IVN fuzzy EDAS technique was selected for its capacity to address uncertainty and ambiguity, rendering it an optimal choice for this intricate evaluation.

The fifth and final step involved an evaluation of the significance of the identified risks and the efficacy of the risk mitigation strategies. This assessment entailed interpreting the findings in the context of the automotive sector, thereby ensuring that the proposed strategies were both relevant and actionable. Table 1 provides a comprehensive account of the 35 identified risks, including their definitions and the literature sources where these risks are mentioned. It is noteworthy that risks numbered 16, 17, 31, and 32 were identified exclusively through expert consultations, which serves to illustrate the value added by expert insights. Table 1 displays 35 separate risks consisting of 5 dimensions: environmental, social, economic, administrative and organizational. These risks were determined through a comprehensive literature review in the field of sustainability in the supply chain. Many of the risks included here are also included in the annual automotive sustainability main industry reports published by some top-tier companies. The study aims to make a new contribution to the literature by adding administrative and organizational dimensions and gathering sustainability under 5 main dimensions. In addition, there are some criteria included in this study for the first time among the SRs in Table 1.

**Table 1:** SRs in the automotive industry.

Environmental Dimension – (Code: R-1)			
Sub-Dimensions	Code	Definition & Risks	Reference
Rising energy usage	SR-1	Energy efficiency is achieved by using energy effectively. It is important for businesses to fulfil some responsibilities for energy efficiency.	[[50], [51], [52]]
Growing levels of greenhouse gas	SR-2	Factors such as car exhausts, smoke from factory chimneys, increasing CO2 and fossil fuels cause an increase in greenhouse gases. Such risks in logistics activities also adversely affect human health.	[22,[52], [53], [54], [55], [56], [57], [58], [59], [60]]
An increase in air, noise, light, and soil contaminants	SR-3	It covers all kinds of pollutants such as light, noise, air, and soil that are harmful to the environment in logistics facilities.	[[52], [53], [54], [55], [56], [57], [58],^60^]
Accidents	SR-4	Accidents that may occur in the supply chain or logistics processes may adversely affect the production flow and cause material and moral losses. These losses may be caused by personnel, machines or business processes.	[22,37,61,62]
Water and Wastewater Risks	SR-5	The increase in water consumption has brought along threats and risks to water resources. Together with the climate crisis, water scarcity poses a threat to the world population.	[59]
Global warming danger and the climate crisis	SR-6	Factors such as ecosystem degradation, extinction of some plant and animal species and global warming have increased the importance of the climate crisis. In particular, the World Economic Forum published a report on combating the climate crisis in 2021.	[63,64]
The frequency of natural catastrophes	SR-7	Natural disasters such as floods, hurricanes, earthquakes, fires, etc. can cause great dangers if necessary precautions are not taken.	[22,37,[65], [66], [67]]
Excessive waste of products	SR-8	Product wastes can occur in all forms and are wastes that do not contribute directly to the product. As a result of the damage it causes to the environment, its negative effects are high.	[56,57,68]
Superfluous packing	SR-9	Factors such as the sensitivity of the products, product content, the distance the product will be transported, and the liquid or solid state of the product constitute important factors in packaging. If efficient packaging is not done, the operation of supply chain processes will be adversely affected.	[22,[55], [56], [57],^69^]
**Social Dimension – (Code: R-2**)			
**Sub-Dimensions**	**Code**	**Definition & Risks**	**Reference**
Failure to maintain work-life balance	SR-10	It involves excessive workloads. The workloads expose individuals to heavy responsibilities which may violate established rules and regulations. As a result, the employee's work-life balance decreases and their motivation deteriorates.	[8,22,37,52]
Unequal pay	SR-11	Wages paid unfairly to employees and wages given as a result of ignoring labor. It occurs as a result of unfair payment policies.	[22,55]
Improper recruitment system	SR-12	These are the risks which arise due to the absence of or inadequate recruitment policies. Inappropriate recruitment and high staff turnover lead to financial and moral loss for enterprises and problems regarding operational efficiency. However, such adverse scenarios can be prevented through the implementation of competency-based interviews.	[8,70,71]
Discrimination (age, religion, language, gender, race, culture, disability, political views)	SR-13	Discrimination in the workplace subsumes mistreating individuals on the grounds of their gender, religion, age, race, culture, political views, disability, or language.	[8,61,70]
Child labor/forced labor status	SR-14	All work that upsets the life balance of children and detrimentally affects their growth is harmful. This includes instances of forced labour beyond the confines of their workplace.	[37,52]
Risks related to the protection of skilled labor	SR-15	It includes layoffs, strikes, protests or demonstrations among workers that manifest as social instability due to unrest, disorder or disruption in the workplace. As a result, employees may continue to cease operations and refrain from purchasing or entering the business.	[22,53,59,70,72,73]
Lack of Information/Data security	SR-16	Unauthorized use and unauthorized disclosure of information or data.	This work
Lack of political security environment, War-terrorist environments	SR-17	Various risks can lead to the infringement of essential human rights and freedoms. For instance, inter-state conflicts arise from war, terrorism, economic, and political challenges, in addition to all kinds of measures aimed at instilling fear in societies.	This work
Lack of employee competence and training	SR-18	It is important to raise awareness of employees with a broad training that includes job-specific or behavioral (personal) competencies. The absence of these elements will have negative consequences.	[59]
Unsafe and unhealthy working environment	SR-19	The lack of regulations concerning occupational health and safety that companies are bound to follow could lead to adverse effects on both workers and the organisation itself. Organizations ought to be open to embracing and integrating occupational health and safety and risk management principles to secure output, efficiency and longevity.	[74]
**Economic – (Code: R-3)**			
**Sub-Dimensions**	**Code**	**Definition & Risks**	**Reference**
High financial risks and volatility in prices	SR-20	Crises resulting from sudden economic losses consist of currency fluctuations, stock market crashes, inflation, and currency fluctuations. The fact that the prices of the products used are variable is the state of constant variability in prices, especially energy price fluctuations.	[22,37,58,69,72]
Risks resulting from low customer satisfaction	SR-21	One of the primary objectives of businesses is to stay afloat. Thus, the company must maximize its earnings. A long-term association with customers is necessary for profit optimization. Customer contentment refers to products or services satisfying the consumers' desires.	[59]
Credit Risk	SR-22	The credit system, also called the direct debit system, is an effective method for securing dealer receivables resulting from domestic vehicle and spare parts sales. As a result of credit risks, problems will arise in the payment methods of the receivables and also cause financial problems.	[64]
Absence of perfect competition environment (bribery, corruption, false statements, false claims)	SR-23	It is a situation of conspiracy between sellers or buyers to coordinate pricing for the mutual benefit of traders. These situations include bribery, corruption, false statements and false claims. For example; Offering (or accepting) money or a gift from a potential customer (from a supplier) in exchange for business	[22,37,72]
**Organizational – (Code: R-4)**			
**Sub-Dimensions**	**Code**	**Definition & Risks**	**Reference**
Machinery and equipment risks (OHS measure)	SR-24	Machinery and equipment may cause injury as they may consist of moving parts. At the same time, breakdowns that may occur in machinery and equipment cause the production line to stop. Such negativities cause disruption of existing works and as a result, deterioration in the organization.	[67,75,76]
Incorrect planning of supply chain processes	SR-25	Planning entails making predictions, setting goals for the future based on these forecasts, and figuring out how to reach those goals. In short, making predictions is an important part of planning. Demand or sales forecasts are especially important for businesses. Different sales and advertising plans are made according to demand forecasts, and stock policies are determined.	[66,67,75,76,80]
Demand Risk: Long and variable product deliveries, Forecast error, Change in consumer preference-	SR-26	The fundamental principle of procurement of goods and services and delivery times in the automotive sector is adherence to sustainable supply chain purchasing regulations. Conducting market research to identify optimal purchasing options is crucial. Extended delivery or maintenance periods, inadequate technical support or spare parts supply, fixed pricing, and pricing guarantees for a limited time span are among the potential risk factors that must be considered.	[63,75,76,78,80]
Supplier uncertainty risks, Supplier error, Procurement commitment, Procurement cost	SR-27	There are important factors that prioritize sustainability in the supply chain, such as monitoring the supplier network and operational competence. Supplier uncertainty risks will cause problems in the internal and external relations of the institution. These may include some applications such as shipping, quality performance, financial standing and capacity adequacy.	[64,75,76,79,81]
Material order risks	SR-28	There are two types of risks that harm the supply chain in the automotive sector: quantitative risks and qualitative risks. Quantitative risks result in a shortage of materials or parts, thus affecting the supply-side activities in the supply chain. Qualitative risks refer to the issues within the supply chain that result in defective parts or materials. This includes inferior quality materials or parts, components with incorrect dimensions, and so on.	[75,76,80,81]
Inventory risks	SR-29	Risk can occur if there is no balance between inventory levels and demands. (There may be an inventory risk if there is no safety stock.) The use of unsuitable vehicles, lack of automation, bad and damaged roads, and unsuitable port facilities are risks.	[[75], [76], [77]]
Transport/Transportation/Transport/Storage risks	SR-30	Transport/Transportation/Transport/Storage risks	[8]
Performance risk	SR-31	Cost, supply assurance, delivery, quality, flexibility, agility.	This work
**Administrative Dimension– (Code: R-5)**			
**Sub-Dimensions**	**Code**	**Definition & Risks**	**Reference**
Lack of commitment and support from top management (managerial)	SR-32	As a result of not performing the work that the management is obliged to fulfill, risks at the social level will occur in the enterprise.	This work
Procurement Management Risks (managerial)	SR-33	Problems in the production of important materials and parts such as steel, plastic, rubber and semiconductors used in automobiles due to the global changes caused by the pandemic and climate change constitute risks in supply management.	[59]
Legal/compliance risk/Reputational risk	SR-34	Legal and compliance risks, and reputational risk as a result of failing to transition to a low-carbon economy. Reputational risk is the loss that may arise due to the loss of confidence in companies by investors, insurance companies and lending institutions or damage to the reputation of companies. It may cause loss of social prestige, decrease in demand for its products and services, and adversely affect competitiveness.	[64]
Technology risk	SR-35	Technological Transition Risk	[64]

In the process of collecting data from experts, different SRs and risk prevention strategies (alternatives) were determined by consulting experts from a leading logistics company in Turkey and using the literature. The linguistic data obtained from the experts were obtained by obtaining opinions from 4 different experts with 10–15 years of experience in the field of sustainability and the logistics sector. Here, the experts have their own opinions, experiences and intuitions. Instead of mathematical and numerical values, descriptive verbal expressions such as high and very high were used in the estimation. This estimation is completely based on subjective judgments. In determining the SR and risk reduction strategies, an extensive literature review and automotive sustainability reports were also used.

Following the risk identification phase, 40 SR strategies were developed. In addition to reviewing articles and automotive sustainability reports, industry experts were consulted in the development of these risk strategies. Accordingly, these alternatives are strategies that could have an impact on one or more of the risks identified in the first phase.

Table 2 shows the strategies (alternatives- AL) developed for SRs. These strategies were created by examining 4 expert opinions and automotive sustainability reports from Turkey's leading logistics company [59,63,64].

**Table 2:** Strategies developed to prevent SRs in the automotive industry (AL: Alterntative).

Code	Definition
AL-1	• To purchase machinery, equipment, and cars with high efficiency. For instance, doing research studies and creation on topics including digitalization, reducing weight, material technologies, self-driving cars, electrification, autonomous driving, fuel alternatives, driver support, and safety systems, as well as interface development for these systems, • Utilizing clean fuel in vehicles used for logistical transportation.
AL-2	• To create technologies for hydrogen or electric vehicles, to put money into charging stations for electric cars, • To introduce new electric car models, • Expanding the product and service offerings, • Opening up new markets and fostering competition, • Lowering reliance on fossil fuels, driving zero- and low-emission vehicles, and being environmentally-friendly.
AL-3	• To execute tasks pertaining to energy efficiency legislation and lowering energy usage, • Reducing the amount of fossil fuels used, utilizing sources of clean energy, and investing in energy-efficient projects, employing the Energy Management System Standards of ISO 50001, • Improving energy efficiency as a means of mitigating the impacts of global warming, • To create innovative, technologically advanced products that are energy and ecologically-friendly while also making efficient use of earth's resources.
AL-4	• To consider the effects of the environment when designing and developing procedures, • Including suppliers in initiatives to reduce greenhouse gas emissions is an illustration.
AL-5	• Putting the facility in a remote region of a city, • To insulation against sound, • To create a sustainable structure that does not display light pollution, • Lowering the impact of flue gas, • Maintaining heating and ventilating systems on a regular basis, • To compile routine reports on the quality of the light, water, air, and soil, • To remove dangerous materials and enhance air quality in activities, new technologies are being developed together with better business practices, • To invest in R&D and creativity in order to lower air pollutants and enhance the performance of cars and other transportation.
AL-6	• Putting together an emergency strategy in case of mishaps, • Delivering ongoing instruction on accident prevention, • Setting objectives and organizing excursions in this regard, • To establish goals and calculate the lost day accident frequency rate (LTAR), seriousness of accident ratio (ASR), and incident frequency rate (TAR), • To plan and construct workspaces in a way that minimizes environmental mishaps.
AL-7	• To ascertain methods for managing and reusing water in businesses' manufacturing processes and to create procedures along these lines, • Cleansing of water from homes produced in treatment plants and wastewater from industries from manufacturing operations.
AL-8	• To lessen the consequences of global warming by making effective use of energy, • To standardize the environmentalist viewpoint by raising knowledge within the organization, • To guarantee that the detrimental effects of goods and activities do not affect the environment or living beings. To comply with the Paris Agreement on mitigating the climate crisis, • To decide on and effectively carry out the business's energy and environmental governance policy, • To establish goals for the battle for global warming, such as cutting emissions in supply chains, operations, and logistics procedures, • Encouraging practices that cut emissions all the way down the supply chain, • To guarantee the EU Green Agreement's transformation of the automotive sector to an environmentally friendly economy.
AL-9	• Creating an emergency strategy and offering instruction in its use, • Using alarms for flame prevention/retardant structures, and seismic isolation devices in structures to protect against earthquakes, • Utilizing smart systems and carrying out research on earthquakes to avert natural disasters, • Carrying out fire and earthquake exercises in companies, • To provide insurance coverage for potential losses in the event of risks within the parameters of the policy, • Putting the company on property that is appropriate and protected from flooding.
AL-10	• Appropriate waste removal or recycling, • To create the goods and employ it in a way that does not damage the natural world, • To create sustainable, ecologically beneficial products, • Optimizing the process subject to practical limitations, • To draft industrial waste disposal strategies for businesses and give maintenance staff the required training, • Endorsing zero-waste initiatives. Lowering the amount of idle capacity.
AL-11	• Packing that is optimized, • To do research and development on package preservation, • Creating packaging that is suitable for the product, • To lessen the quantity of products packaged and the basic components sourced from suppliers involved in the green package project in order to raise awareness of ecological problems and cut down on single-use plastics, • Purchasing packaging made of recycled materials.
AL-12	• To manage the working hours of employees within the framework of labor law and ethical rules,. • Organizing incentive practices such as rewards to increase employee motivation, and ensuring that employees participate in volunteer activities or training during working hours, • To increase productivity in the workplace by executing strategies focused on employee engagement and stakeholder participation, • To ensure that the institution takes actions that increase the personal and professional development of its employees, • Ensuring employee morale and motivation; it consists of issues such as private health insurance, private life insurance, bonuses, graduate scholarships, innovative internship programs, foreign language support, leave entitlement when necessary, and task-based technical training.
AL-13	• To implement a policy of equal pay for equal work, • To end unfair payment and unfair promotion situations.
AL-14	• To create an employment culture related to strategic goals and sustainable priorities, • Establishing appropriate recruitment policy, • To prevent the use of labor other than those specified by law.
AL-15	• Conducting social accountability and social compliance audits of the company, • As a result of these standards, after the suppliers or customers are inspected and their deficiencies are identified, we decide whether or not to work with the supplier/customer.
AL-16	• To create and maintain a fair, equal and appropriate working environment for all employees, customers and suppliers, regardless of race, language, religion, culture, gender, age, disability, political opinion, etc.
AL-17	• Companies develop legal compliance procedures and programs and comply with existing regulations/guidelines, • To adopt the Universal Declaration of Human Rights as a global guide for a healthy working environment, • Not tolerating child labor and all kinds of discrimination. For examle, regulation/principles for managing business disciplines in accordance with occupational health and safety regulations and principles, European Green Agreement, Paris Agreement, Global Risks Report published by the World Economic Forum, Regulation on Monitoring and Reporting of Greenhouse Gas Emissions, etc.
AL-18	• To handle multi-dimensional talent management and the supply and development of a qualified workforce, • To base an education system supported by lifelong learning policies in line with the needs of the industry, with a curriculum suitable for current needs (i.e., data science, cyber security/information security, artificial intelligence, etc.) and to ensure the coordination of this system with public institutions and organizations and the private sector, • To create a highly productive work culture that will maximize employees' competencies such as team building, being result-oriented, analytical thinking and decision-making.
AL-19	• To implement protection policies for the security of personal data and information within the institution, • Using cloud platform for data management. For example, companies conduct sensitivity/penetration tests for information security, • To protect the company and employees against internal and external threats with information security and cyber security measures, and to provide training in these areas.
AL-20	• Compliance with sustainability laws by carrying out all risk identification, risk assessment, risk analysis and the latest risk measures for the institution, especially in the environment of political insecurity, • To make changes in problematic locations of the institution in cases of war and terrorism.
AL-21	• Creating professional and personal training programs for employees.
AL-22	• Collaborating with nearby industries. For example, university-industry cooperation is a choice to improve R&D and innovation capability, • To increase product development capacity, • Collaborating on digital transformation issues.
AL-23	• Taking precautions in case of a possible economic crisis or price fluctuations (e.g., investing in efficiency and savings projects, finding and developing sustainable financing methods).
AL-24	• Executing policies to meet customer needs, • Checking customer feedback systems, • Organizing various surveys, • To follow rival companies closely, • To pursue the digitalization and technological developments of the institutions and to employ employees who will meet customer expectations in the face of increasing technology and digitalization in the automotive industry, • Evaluating the brand's purchasing/after-sales services, examining processes such as marketing and determining customer expectations.
AL-25	• For credit risk, to secure the collection of domestic and international vehicle sales with letters of credit, letters of guarantee, export receivable insurance, bank limit or cash payment methods.
AL-26	• Companies establishing and implementing ethical principles procedures, • To take precautions against potential human rights violations (for full competition).
AL-27	• Gaining a larger portion of the market. Establishing new markets, • To adapt to evolving client needs by providing cutting-edge goods, services, and procedures, • To obtain a competitive edge, • Maintaining the best possible level of consumer fulfillment and to increase organizational efficiency, • To further pursue cutting-edge research and development (R&D) on electrification, connectivity, self-driving cars, electric vehicles, smart manufacturing methods, user experience enhancement, digitization, and intelligent mobility options in to promptly adapt to the industry's technological shift and shifting customer preferences in the automotive sector, • Spreading the company's innovation culture, gather knowledge, and impart acquired experiences by incorporating staff members' creative ideas into our operations through workshops.
AL-28	• Establishing a committee for occupational safety and health, offering training in this area, and making sure businesses abide by all laws, rules, and regulations pertaining to these matters. These committees maintain a safe and healthy work environment by routinely monitoring the organization's present process, • Using risk analysis as a means of preventing hazardous events at work, • To conduct investigations to determine the underlying causes of occupational illnesses and job mishaps, • Following up and providing updates to emergency teams, • Carrying out routine maintenance on devices and machinery, • Providing recurring medical treatment to staff members, • To offer medical services that are preventive and protective. Strict hygienic procedures must be implemented in offices and manufacturing areas.
AL-29	• To effectively manage the flow of materials, equipment, information and money in logistics and supply chain processes.
AL-30	• To carry out logistics and supply chain processes with effective planning and scheduling, • To make the institution a competitive advantage by using effective, efficient and smart systems at every stage such as logistics systems, production, planning and materials. For example; Combining technologies including the “Manufacturing Internet”, “Cyber/Physical Techniques”, “Internet of Things” and “Smart Industries”, • To ensure the correct management of management practices, external relations, organization and information systems within the logistics process, • To make improvements by reviewing the deficiencies in logistics planning with an agile working approach.
AL-31	• To be able to respond to the demand and supply amount in supply chain processes in short intervals, • Planning demand forecasts in the long and short term, • To execute supply chain buying operations in compliance with sustainable handling and purchasing guidelines, • Providing consultancy with live support.
AL-32	• To include sustainability criteria in the evaluations made in supplier selection, • To follow policies to ensure speed, quality, price and flexibility superiority that creates added value in supply chain processes, • Prioritizing sustainability in the supply chain, • Including supplier network traceability, operational competence and crisis management, • Conducting supply risk management appropriately, • To determine the supplier selection and evaluation criteria and to make effective supplier selection with various decision-making methods at the appropriate supplier selection stage, • Improving supplier relationship management, • Making warning and development programs (when deemed necessary) for suppliers whose low performance is determined during audits. Monitoring the company's goals in areas where audits currently show low performance.
AL-33	• Developing production flow management with various modules, • To ensure that the materials supplied are of high quality, • Suppliers sending correct and appropriate materials, • Supplier's ability to provide complete and effective operations such as technical drawings and material-technical documents, • Critical materials with long lead times are not dependent on a single supplier, and at the same time, suppliers of critical materials do not change frequently, • Timely supply of materials, • Flexible layouts for free material flow, • Effective execution of material, information and financial flows.
AL-34	• Developing an effective stock and inventory management system, • To control inventory and stocks that negatively affect companies.
AL-35	• Optimizing transportation types and using appropriate vehicles, • Using appropriate facilities, • Increasing electric freight transportation in logistics, • High availability of intermodal transportation, • Supporting transportation with digital processes.
AL-36	• Carrying out a separate evaluation of the risk associated with suppliers, including factors such as delivery, product quality, adaptability, capacity adequateness, and financial position, and taking appropriate action.
AL-37	• Establishing upper management's objectives and pledges in relation to sustainable objectives, • Presenting preparations for the continuation of operations and business.
AL-38	• To create a supply chain and logistics management system that is sustainable, • To assure our nation's development within the evolving automotive sector and to make it a stronger competitor.
AL-39	• Investing in initiatives that will provide strategic benefit, • Catching opportunities at an early stage, • Collaborating with the entrepreneurship ecosystem, • To guarantee that risks related to compliance and law are handled as carefully as possible, • To determine the company's carbon intensity, Scopes 1 & 2 emissions transition path compared to the sector group and similar companies in the sector with Trucost analysis, • Evaluating the Climate Strategy Score (S&P Global SAM), • Providing digitalization, electrification and smart mobility, • Executives on the board of directors should be routinely informed by the Legal and Compliance Directorate about important disputes and compliance issues and policy decisions faced by the company (e.g., early risk detection, compliance policy, anti-corruption, etc.).
AL-40	• To reduce the negative effects of climate change by disabling existing products and technologies within the scope of technology risk, • Transitioning to low-emission technologies in products and services, • Carrying out Industry 4.0 studies.

**Fig. 2:**
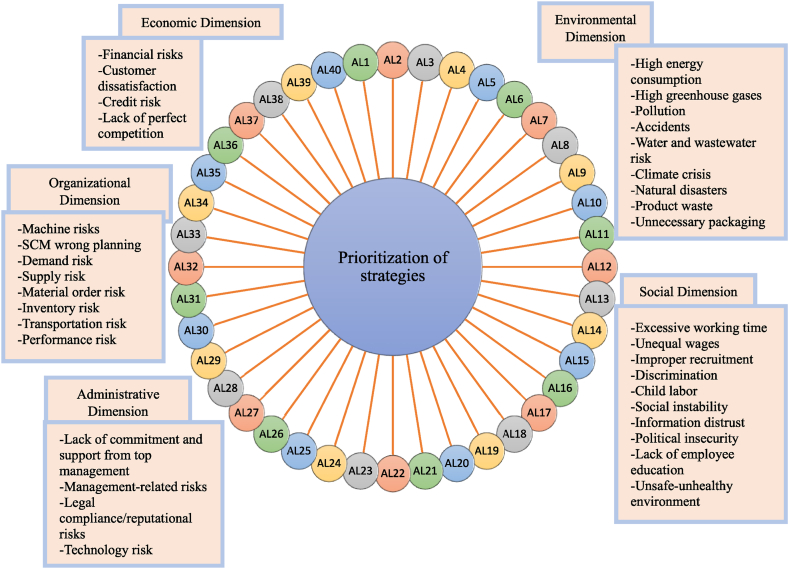
Hierarchy of the proposed method.

As depicted in Fig. 2, the study on SRs in the automotive industry identified 35 SRs and 40 risk strategies (alternatives). The study also incorporates two new dimensions to the sustainability dimensions, thereby expanding the scope of the research. The sustainability dimensions comprise 5 dimensions: environmental, social, economic, managerial, and organizational. Each of the 5 dimensions has sub-criteria, illustrated in Fig. 2.

### 3.1 Interval-valued neutrosophic fuzzy EDAS

The distances between the normalized parameter values and the average solution serve as the foundation for the EDAS approach. The distances between positive and negative ideal solutions are computed using the average solution. Using these best selections allows you to select the ideal solution. The Negative Distance from Average (NDA) indicates the predicted deviation between the average and negative solutions. The predicted deviation between the average and positive solutions is shown by PDA which stands for Positive Distance from Average. We need to find the options with the least negative and most positive deviations from the mean in order to determine which is the best option. Neutrosophic sets are utilized for the first time to popularize the EDAS procedure. Karasan et al. [82] used neutrosophic sets to account for DMs' opinions and tendencies, and the DM's T (Truthness), F (Falsity), and I (Indeterminacy) can be detected.

Definition 1 Let $x_{j}$ = ⟨[*T* $\begin{array}{l} L\\ j \end{array}$, T $\begin{array}{l} U\\ j \end{array}$], [*I* $\begin{array}{l} L\\ j \end{array}$, I $\begin{array}{l} \;U\\ j \end{array}$], [*F* $\begin{array}{l} L\\ j \end{array}$, F $\begin{array}{l} U\\ j \end{array}$]⟩ indicate to a set of IVN, while j pertains to the deciders (j=1,2,…,n). The calculation of the IVN Weighted Arithmetic Average (INWAA) operator can be achieved by Equation (1). This operator is based on the weighted aggregation operators of IVNNs:

(1) $$\left(x_{1},x_{2},\ldots ,x_{n}\right)={\sum_{k=1}^{n}y}_{k}x_{j}=$$

$$\left\langle \left[1-\prod_{k=1}^{n}{\left(1-T\begin{array}{l} L\\ j \end{array}\right)}^{y_{k}},1-\prod_{k=1}^{n}{\left(1-T\begin{array}{l} U\\ j \end{array}\right)}^{y_{k}}\right],\left[\prod_{k=1}^{n}{\left(I\begin{array}{l} L\\ j \end{array}\right)}^{y_{k}},\prod_{k=1}^{n}{\left(I\begin{array}{l} U\\ j \end{array}\right)}^{y_{k}}\right],\left[\prod_{k=1}^{n}{\left(F\begin{array}{l} L\\ j \end{array}\right)}^{y_{k}},\prod_{k=1}^{n}{\left(F\begin{array}{l} U\\ j \end{array}\right)}^{y_{k}}\right]\right\rangle \text{,}$$

where variable $y_{k}$ represents the weight assigned to the DM, indicating its level of importance.

Definition 2 The ranking operation of IVNN can be determined using Equation (2):

(2) $$K\left(x\right)=\frac{\left(T\begin{array}{l} L\\ x\; \end{array}\left(2-I\begin{array}{l} L\\ x \end{array}-I\begin{array}{l} U\\ x \end{array}\right)\right)+\left(T\begin{array}{l} U\\ x \end{array}\;\left(2-I\begin{array}{l} L\\ x \end{array}-I\begin{array}{l} U\\ x \end{array}\right)\right)+\left(1-F\begin{array}{l} L\\ x \end{array}\right)\left(2-I\begin{array}{l} L\\ x \end{array}-I\begin{array}{l} U\\ x \end{array}\right)+\left(1-F\begin{array}{l} U\\ x \end{array}\right)\left(2-I\begin{array}{l} L\\ x \end{array}-I\begin{array}{l} U\\ x \end{array}\right)}{8}\text{,}$$

$$x=\left\langle \left[T\begin{array}{l} L\\ x \end{array},T\begin{array}{l} U\\ x \end{array}\right],\left[I\begin{array}{l} L\\ x \end{array},I\begin{array}{l} U\\ x \end{array}\right],\left[F\begin{array}{l} L\\ x \end{array},F\begin{array}{l} U\\ x \end{array}\right]\right\rangle \text{.}$$

Definition 3 To find the greatest value between the zero and IVN set, Equation (3) is applied:

(3) $$Z\left(x_{j}\right)=\left\{ \begin{array}{c} x_{j},\text{if}\;K\left(x_{j}\right)>0,\\ 0,\text{if}\;K\left(x_{j}\;\right)\leq 0, \end{array}\right.$$

where $x=\left\langle \left[T\begin{array}{l} L\\ j \end{array},T\begin{array}{l} U\\ j \end{array}\right],\left[I\begin{array}{l} L\\ j \end{array},I\begin{array}{l} U\\ j \end{array}\right],\left[F\begin{array}{l} L\\ j \end{array},F\begin{array}{l} U\\ j \end{array}\right]\right\rangle$ and 0=⟨[0,0],[1,1],[1,1]⟩.

Definition 4 Let X be a universe of discourse. An IVN set N in X can be defined independently by a truth membership function $T_{N}\left(x\right)$. An indeterminacy membership function $I_{N}\left(x\right)$ and a falsity membership function $F_{N}\left(x\right)$ for each x∈X: $T_{N}=\left[T\begin{array}{l} L\\ N\left(x\right) \end{array},T\begin{array}{l} U\\ N\left(x\right) \end{array}\subseteq \left[0,1\right]\right],I_{N}\left(x\right)=\left[I\begin{array}{l} L\\ N\left(x\right) \end{array},I\begin{array}{l} U\\ N\left(x\right) \end{array}\subseteq \left[0,1\right]\right]$ and $F_{N}\left(x\right)=\left[F\begin{array}{l} L\\ N\left(x\right) \end{array},F\begin{array}{l} U\\ N\left(x\right) \end{array}\subseteq \left[0,1\right]\right]\text{.}$

This IVN set must satisfy the condition of $0\leq T\begin{array}{l} L\\ N\left(x\right) \end{array}+I\begin{array}{l} L\\ N\left(X\right) \end{array}+F\begin{array}{l} L\\ N\left(x\right) \end{array}\leq 3$.

The IVNS can be expressed as

(4) $$N=\left\{\left\langle x,\left[T\begin{array}{l} L\\ N\left(x\right) \end{array},T\begin{array}{l} U\\ N\left(x\right) \end{array}\right],\left[I\begin{array}{l} L\\ N\left(x\right) \end{array},I\begin{array}{l} U\\ N\left(x\right) \end{array}\right],\left[F\begin{array}{l} L\\ N\left(x\right) \end{array},F\begin{array}{l} U\\ N\left(x\right) \end{array}\right]\right\rangle |\;x\in X\right\}\text{.}$$

Although IVNS can be expressed as Equation (4). The IVNS is denoted as $\left[T\begin{array}{l} L\\ N \end{array},T\begin{array}{l} U\\ N \end{array}\right],\left[I\begin{array}{l} L\\ N \end{array},I\begin{array}{l} U\\ N \end{array}\right],\left[F\begin{array}{l} L\\ N \end{array},F\begin{array}{l} U\\ N \end{array}\right]$ for reducing complexity.

Let $\left[T\begin{array}{l} L\\ a \end{array},T\begin{array}{l} U\\ a \end{array}\right],\left[I\begin{array}{l} L\\ a \end{array},I\begin{array}{l} U\\ a \end{array}\right],\left[F\begin{array}{l} L\\ a \end{array},F\begin{array}{l} U\\ a \end{array}\right]$ and $b=\left[T\begin{array}{l} L\\ b \end{array},T\begin{array}{l} U\\ b \end{array}\right],\left[I\begin{array}{l} L\\ b \end{array},I\begin{array}{l} U\\ b \end{array}\right],\left[F\begin{array}{l} L\\ b \end{array},F\begin{array}{l} U\\ b \end{array}\right]$ be two IVNs. Their relationships are described below:

I. $a^{c}=\left\langle \left[T\begin{array}{l} L\\ a \end{array},T\begin{array}{l} U\\ a \end{array}\right],\left[1-I\begin{array}{l} L\\ a \end{array},1-I\begin{array}{l} U\\ a \end{array}\right],\left[F\begin{array}{l} L\\ a \end{array},F\begin{array}{l} U\\ a \end{array}\right]\right\rangle \text{,}$
II. a⊆b if and only if a⊆b and only if $T\begin{array}{l} L\\ a \end{array}\leq T\begin{array}{l} L\\ b \end{array},T\begin{array}{l} U\\ a \end{array}\leq T\begin{array}{l} U\\ b \end{array};I\begin{array}{l} L\\ a \end{array}\geq I\begin{array}{l} L\\ b \end{array},I\begin{array}{l} U\\ a \end{array}\geq I\begin{array}{l} U\\ b \end{array};\;F\begin{array}{l} L\\ a \end{array}\geq F\begin{array}{l} L\\ b \end{array},F\begin{array}{l} U\\ a \end{array}\geq F\begin{array}{l} U\\ b \end{array};\;a=b$ if and only if a⊆b and b⊆a,
III. $a⨁b=\left\langle \left[T\begin{array}{l} L\\ a \end{array}+T\begin{array}{l} L\\ b \end{array}-T\begin{array}{l} L\\ a \end{array}\;T\begin{array}{l} L\\ b \end{array},T\begin{array}{l} U\\ a \end{array}+T\begin{array}{l} U\\ b \end{array}-T\begin{array}{l} U\\ a \end{array}T\begin{array}{l} U\\ b \end{array}\right],\left[I\begin{array}{l} L\\ a \end{array}I\begin{array}{l} L\\ b \end{array},I\begin{array}{l} U\\ a \end{array}\;I\begin{array}{l} U\\ b \end{array}\right],\left[F\begin{array}{l} L\\ a \end{array}F\begin{array}{l} L\\ b \end{array},F\begin{array}{l} U\\ a \end{array}F\begin{array}{l} U\\ b \end{array}\right]\right\rangle \text{,}$
IV. $a⊗b=\left\langle \left[T\begin{array}{l} L\\ a \end{array}\;T\begin{array}{l} L\\ b \end{array},T\begin{array}{l} U\\ a \end{array}\;T\begin{array}{l} U\\ b \end{array}\right],\left[I\begin{array}{l} L\\ a \end{array}+I\begin{array}{l} L\\ b \end{array}-I\begin{array}{l} L\\ a \end{array}\;I\begin{array}{l} L\\ b \end{array},I\begin{array}{l} U\\ a \end{array}+I\begin{array}{l} U\\ b \end{array}-I\begin{array}{l} U\\ a \end{array}I\begin{array}{l} U\\ b \end{array}\right],\left[F\begin{array}{l} L\\ a \end{array}+F\begin{array}{l} L\\ b \end{array}-F\begin{array}{l} L\\ a \end{array}\;F\begin{array}{l} L\\ b \end{array},F\begin{array}{l} U\\ a \end{array}+F\begin{array}{l} U\\ b \end{array}-F\begin{array}{l} U\\ a \end{array}F\begin{array}{l} U\\ b \end{array}\right]\right\rangle \text{.}$

Definition 5 The subtraction of IVN sets is calculated using Equation (5):

(5) $$x⊖y=\left\langle \left[T\begin{array}{l} L\\ x \end{array}-T\begin{array}{l} U\\ y \end{array},T\begin{array}{l} U\\ x \end{array}-T\begin{array}{l} L\\ y \end{array}\right],\left[\max\left(I\begin{array}{l} L\\ x \end{array},I\begin{array}{l} L\\ y \end{array}\right),\max\left(I\begin{array}{l} U\\ x \end{array},I\begin{array}{l} U\\ y \end{array}\right)\right],\left[F\begin{array}{l} L\\ x \end{array}-F\begin{array}{l} U\\ y \end{array},F\begin{array}{l} U\\ x \end{array}-F\begin{array}{l} L\\ y \end{array}\right]\right\rangle \text{,}$$

where $x=\left\langle \left[T\begin{array}{l} L\\ x \end{array},T\begin{array}{l} U\\ x \end{array}\right],\left[I\begin{array}{l} L\\ x \end{array},I\begin{array}{l} U\\ x \end{array}\right],\left[F\begin{array}{l} L\\ x \end{array},F\begin{array}{l} U\\ x \end{array}\right]\right\rangle \;\text{and}\;y=\left\langle \left[T\begin{array}{l} L\\ y \end{array},T\begin{array}{l} U\\ y \end{array}\right],\left[I\begin{array}{l} L\\ y \end{array},I\begin{array}{l} U\\ y \end{array}\right],\left[F\begin{array}{l} L\\ y \end{array},F\begin{array}{l} U\\ y \end{array}\right]\right\rangle \text{.}$

Definition 6 Let $A=\left\langle \left[T^{L},T^{U}\right],\left[I^{L},I^{U}\right],\left[F^{L},F^{U}\right]\right\rangle$ be an IVN number. The deneutrosophicated value of A is calculated by Equation (6):

(6) $$K\left(A\right)=\frac{T^{L}+T^{U}+2-F^{L}-F^{U}+T^{L}\times T^{U}+\sqrt{\left(1-F^{L}\right)\times \left(1-F^{U}\right)}}{6}\times \left[\left(1-\frac{I^{L}+I^{U}}{2}\right)-(\sqrt{I^{L}\times I^{U}}\right]\text{.}$$

### 3.2 Interval valued neutrosophic EDAS method

**Fig. 3:**
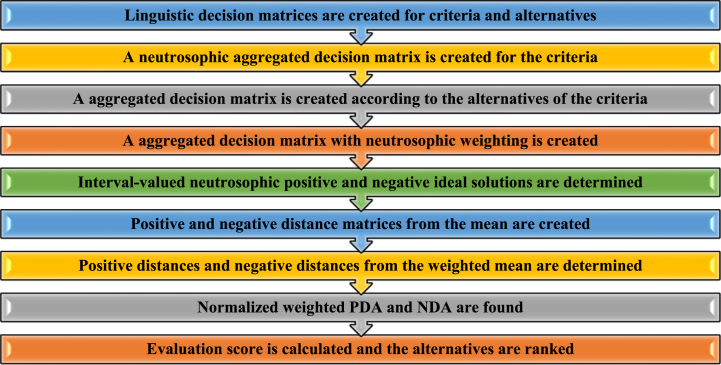
IVN fuzzy EDAS method's stages.

Fig. 3 illustrates the sequential stages of the IVN fuzzy EDAS approach. The initial step involves establishing a linguistic decision matrix for the criteria and alternatives, with nine specific linguistic terms formulated based on expert input. In the subsequent step, the decision matrices are transformed into a neutrosophic format, resulting in the creation of combined and weighted decision matrices. The neutrosophic definition formulas will be used in the numerical operations conducted in this study. After completing all steps, the alternatives will be ranked based on the final evaluation score.

The stages of the IVN fuzzy EDAS approach are illustrated in this section. Upon completion of the steps, the options can be assessed [83].

Step 1 Initially, the IVN decision matrix *D*_*j*_ is created based on expert opinions (j)*,* in which *b*_*mn*_ and *c*_*mn*_ represent the benefit and cost variables, subsequently. The columns indicate alternatives (n)*,* and the rows signify criteria (m). A scale is created that includes linguistic phrases and their associated values in order to build the IVN decision matrix [83]. Table 3 illustrates the IVN decision matrix scales and linguistic terms employed to create the matrix. Finally, the IVN decision matrix (*D*_*j*_) containing linguistic terms is converted to IVN numbers. There is a certain rule in creating Table 3. For example, the lower part of T represents a certain increase from CL to CH with a range of 0.10 from 0.15 to 0.85. Similarly, the lower part of F stands for a decrease from CL to CH with a range of 0.10 from 0.85 to 0.15 [83].

**Table 3:** IVN Decision matrix scale.

Linguistic Terms		⟨T, I, F⟩
CL	Certainly Low	⟨[0.15, 0.25], [0.15, 0.25], [0.85, 0.95]⟩
VL	Very Low	⟨[0.25, 0.35],[0.35, 0.45], [0.75, 0.85]⟩
L	Low	⟨[0.35, 0.45], [0.45, 0.55], [0.65, 0.75]⟩
BA	Below Average	⟨[0.45, 0.55], [0.55, 0.65], [0.55, 0.65]⟩
A	Average	⟨[0.55, 0.60], [0.65, 0.75], [0.45, 0.55]⟩
AA	Above Average	⟨[0.55, 0.65], [0.55, 0.65], [0.45, 0.55]⟩
H	High	⟨[0.65, 0.75], [0.45, 0.55], [0.35, 0.45]⟩
VH	Very High	⟨[0.75, 0.85], [0.25, 0.35], [0.25, 0.35]⟩
CH	Certainly High	⟨[0.85, 0.95], [0.15, 0.25], [0.15, 0.25]⟩

Step 2 To determine the mean, combine all decision matrices to create the IVN decision matrix. The methodology for achieving the average IVN decision matrix is outlined in Table 4. To create the average IVN decision matrix, the formulas in Definition 1 are used. Here, the formula in definition 1 is used for each alternative.

**Table 4:** IVN matrix with criteria and alternatives.

Criterion	Type	*AL*_1_	*AL*_2_	*…*	*AL*_*N*_
*C*_11_	Linguistic Cost	$\left\langle \left[T_{j}^{L},T_{j}^{U}\right],\left[I_{j}^{L}{,I}_{j}^{U}\right],\left[F_{j}^{L},F_{j}^{U}\right]\right\rangle$	$\left\langle \left[T_{j}^{L},T_{j}^{U}\right],\left[I_{j}^{L}{,I}_{j}^{U}\right],\left[F_{j}^{L},F_{j}^{U}\right]\right\rangle$	*…*	$\left\langle \left[T_{j}^{L},T_{j}^{U}\right],\left[I_{j}^{L}{,I}_{j}^{U}\right],\left[F_{j}^{L},F_{j}^{U}\right]\right\rangle$
*C*_12_	Numerical Cost	$\left\langle \left[T_{j}^{L},T_{j}^{U}\right],\left[I_{j}^{L}{,I}_{j}^{U}\right],\left[F_{j}^{L},F_{j}^{U}\right]\right\rangle$	$\left\langle \left[T_{j}^{L},T_{j}^{U}\right],\left[I_{j}^{L}{,I}_{j}^{U}\right],\left[F_{j}^{L},F_{j}^{U}\right]\right\rangle$	*…*	$\left\langle \left[T_{j}^{L},T_{j}^{U}\right],\left[I_{j}^{L}{,I}_{j}^{U}\right],\left[F_{j}^{L},F_{j}^{U}\right]\right\rangle$
⋮	⋮	⋮	⋮	⋮	⋮
*C*_*mn*_	Linguistic Benefit	$\left\langle \left[T_{j}^{L},T_{j}^{U}\right],\left[I_{j}^{L}{,I}_{j}^{U}\right],\left[F_{j}^{L},F_{j}^{U}\right]\right\rangle$	$\left\langle \left[T_{j}^{L},T_{j}^{U}\right],\left[I_{j}^{L}{,I}_{j}^{U}\right],\left[F_{j}^{L},F_{j}^{U}\right]\right\rangle$	*…*	$\left\langle \left[T_{j}^{L},T_{j}^{U}\right],\left[I_{j}^{L}{,I}_{j}^{U}\right],\left[F_{j}^{L},F_{j}^{U}\right]\right\rangle$

Step 3 The average solution matrix of the criterion weights is created by the experts. Using Equation (1), the weights, *W =* [*w*_*j*_]_1×*m*_*,* are determined. The DMs or experts assign linguistic words of weights for each criterion using the IVN criteria weights. Linguistic terms are converted into IVN numbers for criterion weights when the IVN weight matrix is created. The IVN numbers for the criterion weights and language words are shown in Table 5. To create the average IVN decision matrix, the formulas in Definition 1 are used. Here, the formula in Definition 1 is used for each criterion.

**Table 5:** IVN aggregated decision matrix.

Criterion	Type	*AL*_1_	*AL*_2_	*…*	*AL*_*N*_
*C*_11_	Linguistic Cost	$\left\langle \left[T_{A}^{L},T_{A}^{U}\right],\left[I_{A}^{L}{,I}_{A}^{U}\right],\left[F_{A}^{L},F_{A}^{U}\right]\right\rangle$	$\left\langle \left[T_{A}^{L},T_{A}^{U}\right],\left[I_{A}^{L}{,I}_{A}^{U}\right],\left[F_{A}^{L},F_{A}^{U}\right]\right\rangle$	*…*	$\left\langle \left[T_{A}^{L},T_{A}^{U}\right],\left[I_{A}^{L}{,I}_{A}^{U}\right],\left[F_{A}^{L},F_{A}^{U}\right]\right\rangle$
*C*_12_	Numerical Cost	$\left\langle \left[T_{A}^{L},T_{A}^{U}\right],\left[I_{A}^{L}{,I}_{A}^{U}\right],\left[F_{A}^{L},F_{A}^{U}\right]\right\rangle$	$\left\langle \left[T_{A}^{L},T_{A}^{U}\right],\left[I_{A}^{L}{,I}_{A}^{U}\right],\left[F_{A}^{L},F_{A}^{U}\right]\right\rangle$	*…*	$\left\langle \left[T_{A}^{L},T_{A}^{U}\right],\left[I_{A}^{L}{,I}_{A}^{U}\right],\left[F_{A}^{L},F_{A}^{U}\right]\right\rangle$
⋮	⋮	⋮	⋮	⋮	⋮
*C*_*mn*_	Linguistic Benefit	$\left\langle \left[T_{A}^{L},T_{A}^{U}\right],\left[I_{A}^{L}{,I}_{A}^{U}\right],\left[F_{A}^{L},F_{A}^{U}\right]\right\rangle$	$\left\langle \left[T_{A}^{L},T_{A}^{U}\right],\left[I_{A}^{L}{,I}_{A}^{U}\right],\left[F_{A}^{L},F_{A}^{U}\right]\right\rangle$	*…*	$\left\langle \left[T_{A}^{L},T_{A}^{U}\right],\left[I_{A}^{L}{,I}_{A}^{U}\right],\left[F_{A}^{L},F_{A}^{U}\right]\right\rangle$

Step 4 The matrix of average criterion weights is created, then we have *AV*_*n*_ *=* <[$T_{AV}^{L},T_{AV}^{U}$]*,* [$I_{AV}^{L},\;I_{AV}^{U}$]*,* [$F_{AV}^{L},\;F_{AV}^{U}$]>. All IVN criteria weights are summed to arrive at the average criteria weight using the IVN weighted arithmetic mean operation Table 5 displays the weights of the criteria. The average weights for the scores are derived from the decision matrix. All operations performed here are performed neutrosophically. For example, in this step, the criterion weight and the decision matrix are multiplied neutrosophically as given in Relationship IV of Definition 4.

Step 5 PDA and NDA formulas according to the benefit and cost criteria are given in Equations (7), (8).

*PDA =* [*pda*_*mn*_]_*m×n*_*, NDA=*[*nda*_*mn*_]_*m×n*_ are the matriceswhere *pda*_*mn*_ and *nda*_*mn*_ represent the performance values of the *n*th alternative's positive and negative distance from the mean solution in terms of the *m*th criterion, respectively. For *PDA*, if the criterion is benefit-oriented, the first expression of Equation (7) is employed, and if it is cost-oriented, the second expression of Equation (7) is used. Likewise, for *NDA*, if the criterion is beneficial, the 1st expression of Equation (8) is applied, and if the criterion is cost-oriented, the 2nd expression of Equation (8) is employed. When calculating *PDA* and *NDA*, first the IVN decision matrix and the average IVN decision matrix are neutrosophically extracted and then divided by $K\left({av}_{n}\right)$. Here, the calculation of K(av) is done according to Equation (2) in Definition 2:

(7) $${pda}_{mn}=\left\{ \begin{array}{c} \frac{Z\left(x_{mn}⊖AVn\right)}{K\left({av}_{n}\right)},\text{if}\;m\in B,\\ \frac{Z\left({AV}_{n}⊖X_{mn}\right)}{K\left({av}_{n}\right)},\text{if}\;m\in C, \end{array}\right.$$

(8) $${nda}_{mn}=\left\{ \begin{array}{c} \frac{Z\left({AV}_{n}⊖X_{mn}\right)}{K\left({av}_{n}\right)},\text{if}\;m\in B,\\ \frac{Z\left(x_{mn}⊖AVn\right)}{K\left({av}_{n}\right)},\text{if}\;m\in C. \end{array}\right.$$

Step 6 Equation (9) shows the *sp*_*n*_ and *np*_*n*_ formulas.

(9) $${sp}_{n}=\sum_{n=1}^{l}\left(w_{j}⊗{pda}_{mn}\right);\;{np}_{n}=\sum_{n=1}^{l}\left(w_{j}⊗{nda}_{mn}\right)\text{,}$$

Step 7 Here, the values of ${sp}_{n}$ and ${np}_{n}$ are normalized. The normalization values of ${sp}_{n}$ and ${np}_{n}$ for all alternatives are respectively computed by Equations (10), (11):

(10) $${nsp}_{n}=\frac{{sp}_{n}}{\max\left(K\left({sp}_{n}\right)\right)}\text{.}$$

(11) $${nsn}_{n}=1-\frac{{sn}_{n}}{\max\left(K\left({sp}_{n}\right)\right)}\text{,}$$

Step 8 The evaluation score is calculated according to Equation (12) for all alternatives.

(12) $$as_{n}=\frac{1}{2}\left({nsp}_{n}⊕{nsn}_{n}\right)\text{,}$$

Step 9 The evaluation scores are ranked in descending order.

## 4 Case study and results

Businesses in the automotive supply network can fall short of expectations in terms of their social, economic, and environmental performances all of which include sustainability dimensions despite the automotive sector being an important one for sustainability. Among the reasons for this are the high SRs of the automotive sector and the constant development of technology in this field. In recent years, the importance of sustainability has increased, and new approaches, new dimensions, and new performance criteria have been developed [84,85]. In this work, managerial and organizational dimensions were added to social, environmental and economic sustainability factors.

This study presents 40 alternatives aimed at reducing SRs in the automotive sector. These measures are comprehensive and cover potential risks effectively. The study evaluates 35 criteria, organized into five dimensions: environmental, social, economic, organizational, and managerial. Criteria weights were determined by four experts from Turkey's leading logistics organization and have a significance level of 0.25. We ranked and prioritized the alternatives using the IVN fuzzy EDAS method. The neurotrophic fuzzy EDAS method provided us with inputs, including sub-criteria and weights determined by expert opinion, for our final decision. The subsequent stages and results of the IVN fuzzy EDAS approach are presented below.

Each DM developed the IVN matrix according to the provided criteria standards, assigning linguistic terms to all possible alternative options. Table 6 presents the initial expert opinions from the four experts involved in the study. The table outlines the criteria in rows and the various alternatives in columns (the complete form can be found in Table A1). DM-1 then reviewed and rated the alternatives based on the criteria. For instance, Alternative 1 was classified as “certinly high” (CH) for Criterion 1. The experts defined the linguistic terms for each criterion value, with a particular focus on the extent to which the alternatives mitigate existing risks. All criteria in this study are evaluated in terms of benefit to ascertain the potential impact of each alternative and identify the most effective risk mitigation strategy. The involvement of experts ensured detailed evaluations of risks and potential mitigation measures, guaranteeing that decisions were based on informed opinions.

**Table 6:** Linguistic decision matrix for DM-1.

K/A	A1	A2	A3	…	A40
**K1**	CH	CH	CH	…	VH
**K2**	CH	CH	VH	…	CH
**K3**	VH	VH	VH	…	VH
**K4**	AA	CL	A	…	H
**…**	…	…	…	…	…
**K35**	VH	VH	VH	…	CH

Decision matrices, which are the first step of the IVN fuzzy EDAS method, are given in the previous section. In the creation of the decision matrices in Step 1, both the criteria weights and the decision matrices of the alternatives depending on the criteria are included. This step was created by the experts with the help of a questionnaire. Therefore, the method will continue from Step 2 in this section.

Step 2 The average IVN decision matrix is determined by summing all IVN decision matrices. Table 7 shows the values of the aggregated IVN decision matrix for the first alternative. The lower and upper T, L and F values of each SR are given in Table 7.

**Table 7:** IVN decision matrix (group-alternative 1).

A/K	TL	TU	IL	IU	FL	FU	A/K	TL	TU	IL	IU	FL	FU
**SR1**	0.728	0.881	0.573	0.674	0.066	0.221	**SR19**	0.367	0.532	0.245	0.356	0.431	0.596
**SR2**	0.728	0.881	0.573	0.674	0.066	0.221	**SR20**	0.426	0.600	0.173	0.283	0.374	0.548
**SR3**	0.678	0.832	0.523	0.624	0.114	0.271	**SR21**	0.583	0.737	0.416	0.518	0.211	0.366
**SR4**	0.443	0.606	0.245	0.356	0.354	0.518	**SR22**	0.202	0.352	0.447	0.548	0.598	0.748
**SR5**	0.407	0.560	0.322	0.423	0.389	0.542	**SR23**	0.202	0.352	0.447	0.548	0.598	0.748
**SR6**	0.728	0.881	0.573	0.674	0.066	0.221	**SR24**	0.577	0.729	0.423	0.523	0.220	0.372
**SR7**	0.250	0.400	0.400	0.500	0.550	0.700	**SR25**	0.413	0.600	0.132	0.238	0.387	0.573
**SR8**	0.401	0.577	0.173	0.283	0.398	0.573	**SR26**	0.250	0.400	0.400	0.500	0.550	0.700
**SR9**	0.401	0.577	0.173	0.283	0.398	0.573	**SR27**	0.280	0.431	0.366	0.468	0.519	0.670
**SR10**	0.101	0.252	0.548	0.648	0.698	0.849	**SR28**	0.427	0.577	0.300	0.400	0.373	0.523
**SR11**	0.050	0.200	0.600	0.700	0.750	0.900	**SR29**	0.413	0.600	0.132	0.238	0.387	0.573
**SR12**	0.101	0.252	0.548	0.648	0.698	0.849	**SR30**	0.465	0.644	0.197	0.313	0.322	0.505
**SR13**	0.101	0.252	0.548	0.648	0.698	0.849	**SR31**	0.443	0.606	0.245	0.356	0.354	0.518
**SR14**	0.150	0.300	0.500	0.600	0.650	0.800	**SR32**	0.302	0.452	0.346	0.477	0.497	0.648
**SR15**	0.205	0.356	0.440	0.542	0.593	0.744	**SR33**	0.438	0.600	0.228	0.336	0.362	0.523
**SR16**	0.176	0.326	0.473	0.573	0.623	0.774	**SR34**	0.503	0.654	0.346	0.447	0.296	0.447
**SR17**	0.230	0.381	0.416	0.518	0.569	0.720	**SR35**	0.704	0.859	0.548	0.648	0.087	0.245
**SR18**	0.253	0.404	0.394	0.495	0.545	0.696

Steps 3–4 Table 8 presents the IVN criteria weights and total IVN numbers, which DMs use to assign linguistic terms for each criterion. Similar to Step 1, the DMs assess the significance of the criteria and determine the linguistic terms only once.

**Table 8:** Linguistic values of the criteria and weighted aggregated decision matrix of criterion.

SR	DM-1	DM-2	DM-3	DM-4	SR	DM-1	DM-2	DM-3	DM-4	SR	TL	TU	IL	IU	FL	FU	C	TL	TU	IL	IU	FL	FU
**SR1**	CH	CH	CH	CH	**SR19**	CH	CH	CH	CH	**SR1**	0.750	0.950	0.600	0.700	0.050	0.250	**SR19**	0.750	0.950	0.600	0.700	0.050	0.250
**SR2**	CH	CH	CH	CH	**SR20**	H	H	AA	A	**SR2**	0.750	0.950	0.600	0.700	0.050	0.250	**SR20**	0.492	0.694	0.263	0.376	0.306	0.508
**SR3**	H	H	AA	AA	**SR21**	H	H	H	H	**SR3**	0.503	0.704	0.346	0.447	0.296	0.497	**SR21**	0.550	0.750	0.400	0.500	0.250	0.450
**SR4**	VH	VH	VH	VH	**SR22**	A	A	BA	A	**SR4**	0.650	0.850	0.500	0.600	0.150	0.350	**SR22**	0.388	0.588	0.132	0.238	0.412	0.612
**SR5**	H	H	H	H	**SR23**	H	H	VH	VH	**SR5**	0.550	0.750	0.400	0.500	0.250	0.450	**SR23**	0.603	0.806	0.447	0.548	0.194	0.397
**SR6**	CH	CH	CH	VH	**SR24**	VH	VH	H	H	**SR6**	0.728	0.934	0.573	0.674	0.066	0.272	**SR24**	0.603	0.806	0.447	0.548	0.194	0.397
**SR7**	VH	VH	H	AA	**SR25**	VH	VH	VH	VH	**SR7**	0.583	0.789	0.416	0.518	0.211	0.417	**SR25**	0.650	0.850	0.500	0.600	0.150	0.350
**SR8**	AA	AA	H	H	**SR26**	AA	AA	A	AA	**SR8**	0.503	0.704	0.346	0.447	0.296	0.497	**SR26**	0.438	0.638	0.228	0.336	0.362	0.562
**SR9**	A	A	A	AA	**SR27**	VH	VH	H	H	**SR9**	0.413	0.613	0.132	0.238	0.387	0.587	**SR27**	0.603	0.806	0.447	0.548	0.194	0.397
**SR10**	H	H	AA	AA	**SR28**	H	H	AA	H	**SR10**	0.503	0.704	0.346	0.447	0.296	0.497	**SR28**	0.527	0.728	0.372	0.473	0.272	0.473
**SR11**	H	H	AA	H	**SR29**	H	H	AA	AA	**SR11**	0.527	0.728	0.372	0.473	0.272	0.473	**SR29**	0.503	0.704	0.346	0.447	0.296	0.497
**SR12**	H	H	H	H	**SR30**	VH	VH	VH	H	**SR12**	0.550	0.750	0.400	0.500	0.250	0.450	**SR30**	0.627	0.830	0.473	0.573	0.170	0.373
**SR13**	H	H	H	H	**SR31**	VH	VH	VH	VH	**SR13**	0.550	0.750	0.400	0.500	0.250	0.450	**SR31**	0.650	0.850	0.500	0.600	0.150	0.350
**SR14**	CH	CH	CH	CH	**SR32**	H	H	VH	CH	**SR14**	0.750	0.950	0.600	0.700	0.050	0.250	**SR32**	0.635	0.853	0.468	0.569	0.147	0.365
**SR15**	H	H	VH	VH	**SR33**	H	H	VH	H	**SR15**	0.603	0.806	0.447	0.548	0.194	0.397	**SR33**	0.577	0.780	0.423	0.523	0.220	0.423
**SR16**	VH	VH	VH	H	**SR34**	VH	VH	VH	H	**SR16**	0.627	0.830	0.473	0.573	0.170	0.373	**SR34**	0.627	0.830	0.473	0.573	0.170	0.373
**SR17**	AA	AA	A	AA	**SR35**	H	H	H	AA	**SR17**	0.438	0.638	0.228	0.336	0.362	0.562	**SR35**	0.527	0.728	0.372	0.473	0.272	0.473
**SR18**	H	H	AA	H						**SR18**	0.527	0.728	0.372	0.473	0.272	0.473

The evaluation process calculates the average weights for each criterion, and DMs select the appropriate linguistic terms. Table 8 digitizes these linguistic expressions, showing the calculated weights for the lower and upper values of criteria T, L, and F.

Steps 5–6 Table 9 presents the *sp*_*n*_ and *np*_*n*_ values for 40 alternatives. The complete form can be found in Table A2. These values are calculated using Equation (9) in Step 6 of the IVN EDAS method, where the *PDA* and *NDA* values and weights are multiplied using neutrosophic logic.

**Table 9:** Values obtained for the *sp*_*n*_ and *np*_*n*_.

Alternatives	*sp*_*n*_						*np*_*n*_					
	TL	TU	IL	IU	FL	FU	TL	TU	IL	IU	FL	FU
**AL1**	−0.081	0.058	0.124	0.190	−0.014	0.063	0.939	1.044	0.906	0.856	0.990	1.048
**AL2**	−0.093	0.041	0.130	0.197	−0.010	0.071	0.929	1.031	0.901	0.851	0.993	1.054
**AL3**	−0.088	0.049	0.127	0.193	−0.012	0.067	0.934	1.037	0.904	0.853	0.991	1.051
**…**	…	…	…	…	…	…	…	…	…	…	…	…
**AL40**	−0.060	0.089	0.114	0.178	−0.024	0.045	0.955	1.067	0.913	0.865	0.982	1.034

Step 7 In Table 10, the *nsp*_*n*_ and *nsn*_*n*_ values of the alternatives are computed based on every criterion wherein the normalized values of *sp*_*n*_ and *np*_*n*_ are calculated. To do so, Equations (10), (11) from Step 7 are employed.

**Table 10:** Normalized values for the *sp*_*n*_ and *np*_*n*_.

Alternatives	*nsp*_*n*_						*nsn*_*n*_					
	TL	TU	IL	IU	FL	FU	TL	TU	IL	IU	FL	FU
**AL1**	−14.076	10.166	21.672	33.154	−2.383	11.003	14.076	−10.166	21.672	33.154	2.383	−11.003
**AL2**	−16.261	7.135	22.719	34.386	−1.725	12.340	16.261	−7.135	22.719	34.386	1.725	−12.340
**AL3**	−15.290	8.614	22.184	33.752	−2.119	11.645	15.290	−8.614	22.184	33.752	2.119	−11.645
**AL4**	−12.328	12.418	22.033	33.598	−3.388	9.154	12.328	−12.418	22.033	33.598	3.388	−9.154
**AL5**	−13.126	11.519	21.654	33.123	−3.087	9.826	13.126	−11.519	21.654	33.123	3.087	−9.826
**AL6**	−10.033	15.900	20.649	31.862	−4.447	7.465	10.033	−15.900	20.649	31.862	4.447	−7.465
**AL7**	−19.065	3.567	22.177	33.826	−1.259	13.623	19.065	−3.567	22.177	33.826	1.259	−13.623
**AL8**	−12.254	13.003	21.233	32.656	−3.482	9.230	12.254	−13.003	21.233	32.656	3.482	−9.230
**AL9**	−16.979	6.025	22.043	33.737	−1.252	12.975	16.979	−6.025	22.043	33.737	1.252	−12.975
**AL10**	−13.506	10.907	21.677	33.208	−3.113	9.846	13.506	−10.907	21.677	33.208	3.113	−9.846
**AL11**	−15.994	7.695	21.145	32.606	−2.596	11.140	15.994	−7.695	21.145	32.606	2.596	−11.140
**AL12**	−12.052	13.379	21.251	32.707	−3.727	8.999	12.052	−13.379	21.251	32.707	3.727	−8.999
**AL13**	−15.478	8.541	22.367	34.023	−2.852	10.811	15.478	−8.541	22.367	34.023	2.852	−10.811
**AL14**	−8.115	18.493	20.759	32.007	−4.677	6.732	8.115	−18.493	20.759	32.007	4.677	−6.732
**AL15**	−5.908	21.628	19.571	30.539	−5.643	5.026	5.908	−21.628	19.571	30.539	5.643	−5.026
**AL16**	−16.715	6.530	23.909	36.006	−1.614	12.582	16.715	−6.530	23.909	36.006	1.614	−12.582
**AL17**	0.275	29.304	21.309	32.659	−8.007	0.127	−0.275	−29.304	21.309	32.659	8.007	−0.127
**AL18**	−7.352	19.828	19.573	30.591	−5.479	5.673	7.352	−19.828	19.573	30.591	5.479	−5.673
**AL19**	−15.574	8.358	20.816	32.231	−1.909	12.031	15.574	−8.358	20.816	32.231	1.909	−12.031
**AL20**	−12.263	13.283	19.766	30.916	−3.704	9.105	12.263	−13.283	19.766	30.916	3.704	−9.105
**AL21**	−7.751	19.387	19.219	30.187	−5.358	5.961	7.751	−19.387	19.219	30.187	5.358	−5.961
**AL22**	−9.943	16.084	19.176	30.130	−4.616	7.289	9.943	−16.084	19.176	30.130	4.616	−7.289
**AL23**	−9.264	17.134	20.216	31.412	−4.972	6.627	9.264	−17.134	20.216	31.412	4.972	−6.627
**AL24**	−11.022	14.694	19.801	30.934	−4.608	7.508	11.022	−14.694	19.801	30.934	4.608	−7.508
**AL25**	−17.709	5.551	21.149	32.623	−2.392	11.802	17.709	−5.551	21.149	32.623	2.392	−11.802
**AL26**	−6.646	21.166	21.743	33.231	−5.619	5.524	6.646	−21.166	21.743	33.231	5.619	−5.524
**AL27**	−11.656	14.312	19.171	30.124	−4.425	8.120	11.656	−14.312	19.171	30.124	4.425	−8.120
**AL28**	−9.865	16.156	20.179	31.308	−4.129	7.848	9.865	−16.156	20.179	31.308	4.129	−7.848
**AL29**	−7.364	19.608	20.660	31.974	−6.371	4.344	7.364	−19.608	20.660	31.974	6.371	−4.344
**AL30**	−3.965	24.478	20.721	31.977	−6.934	2.993	3.965	−24.478	20.721	31.977	6.934	−2.993
**AL31**	−13.598	11.139	21.073	32.444	−3.785	9.164	13.598	−11.139	21.073	32.444	3.785	−9.164
**AL32**	−7.547	19.144	20.104	31.194	−5.595	5.298	7.547	−19.144	20.104	31.194	5.595	−5.298
**AL33**	−10.223	16.174	20.319	31.545	−5.106	6.979	10.223	−16.174	20.319	31.545	5.106	−6.979
**AL34**	−11.416	14.340	20.307	31.489	−4.605	7.662	11.416	−14.340	20.307	31.489	4.605	−7.662
**AL35**	−10.959	14.992	20.326	31.469	−4.039	8.444	10.959	−14.992	20.326	31.469	4.039	−8.444
**AL36**	−11.386	14.663	19.682	30.735	−4.477	8.093	11.386	−14.663	19.682	30.735	4.477	−8.093
**AL37**	−0.574	28.294	21.366	32.757	−8.336	−0.098	0.574	−28.294	21.366	32.757	8.336	0.098
**AL38**	−7.308	19.695	19.583	30.612	−5.952	4.919	7.308	−19.695	19.583	30.612	5.952	−4.919
**AL39**	−6.521	21.418	19.930	31.023	−5.811	5.287	6.521	−21.418	19.930	31.023	5.811	−5.287
**AL40**	−10.392	15.451	19.973	31.064	−4.185	7.936	10.392	−15.451	19.973	31.064	4.185	−7.936

Step 8–9 In Table 11, evaluation scores are calculated for each alternative. Step 8 uses Equation (6) to calculate the scores, which are then arranged in descending order. This normalized value is computed independently for each alternative and is defined as an evaluation point. Accordingly, Alternative 17 with a value of 0.717 ranks first. Alternative 7 ranks last with a value of 0.679.

**Table 11:** Evaluation scores.

Alternatives	TL	TU	IL	IU	FL	FU	Score	Alternatives	TL	TU	IL	IU	FL	FU	Score
**AL1**	0.467	0.521	0.056	0.081	−0.007	0.033	0.68947	**AL21**	0.482	0.537	0.050	0.075	−0.015	0.018	0.70696
**AL2**	0.461	0.515	0.059	0.084	−0.005	0.037	0.68357	**AL22**	0.477	0.532	0.050	0.075	−0.013	0.022	0.70306
**AL3**	0.464	0.518	0.057	0.083	−0.006	0.035	0.68652	**AL23**	0.479	0.534	0.053	0.078	−0.014	0.020	0.70264
**AL4**	0.471	0.525	0.057	0.082	−0.010	0.027	0.69282	**AL24**	0.475	0.529	0.052	0.077	−0.013	0.022	0.70051
**AL5**	0.469	0.523	0.056	0.081	−0.009	0.029	0.69197	**AL25**	0.458	0.512	0.055	0.080	−0.007	0.036	0.68544
**AL6**	0.477	0.531	0.054	0.079	−0.012	0.022	0.70003	**AL26**	0.485	0.540	0.056	0.081	−0.016	0.016	0.70411
**AL7**	0.454	0.508	0.057	0.083	−0.004	0.041	0.6798	**AL27**	0.473	0.529	0.050	0.075	−0.012	0.024	0.70063
**AL8**	0.472	0.526	0.055	0.080	−0.010	0.027	0.69453	**AL28**	0.477	0.532	0.053	0.077	−0.012	0.023	0.7006
**AL9**	0.460	0.513	0.057	0.082	−0.004	0.039	0.6829	**AL29**	0.483	0.538	0.054	0.079	−0.018	0.013	0.70634
**AL10**	0.468	0.522	0.056	0.081	−0.009	0.029	0.6914	**AL30**	0.491	0.546	0.054	0.079	−0.019	0.009	0.71125
**AL11**	0.462	0.516	0.055	0.080	−0.007	0.033	0.68812	**AL31**	0.468	0.523	0.055	0.080	−0.011	0.027	0.69348
**AL12**	0.472	0.527	0.055	0.080	−0.011	0.027	0.69508	**AL32**	0.483	0.537	0.053	0.077	−0.016	0.016	0.70601
**AL13**	0.463	0.518	0.058	0.083	−0.008	0.032	0.6871	**AL33**	0.477	0.532	0.053	0.078	−0.014	0.021	0.70135
**AL14**	0.482	0.536	0.054	0.079	−0.013	0.020	0.70262	**AL34**	0.474	0.529	0.053	0.078	−0.013	0.023	0.69915
**AL15**	0.487	0.541	0.051	0.076	−0.016	0.015	0.70909	**AL35**	0.475	0.530	0.053	0.078	−0.011	0.025	0.69876
**AL16**	0.460	0.514	0.061	0.087	−0.005	0.038	0.68058	**AL36**	0.474	0.529	0.052	0.076	−0.013	0.024	0.70005
**AL17**	0.501	0.553	0.055	0.080	−0.022	0.000	0.71668	**AL37**	0.499	0.551	0.056	0.080	−0.023	0.000	0.71613
**AL18**	0.483	0.538	0.051	0.076	−0.015	0.017	0.707	**AL38**	0.483	0.538	0.051	0.076	−0.017	0.014	0.70778
**AL19**	0.463	0.517	0.054	0.079	−0.005	0.036	0.68814	**AL39**	0.485	0.541	0.052	0.077	−0.016	0.015	0.70789
**AL20**	0.472	0.527	0.052	0.077	−0.010	0.027	0.69751	**AL40**	0.476	0.531	0.052	0.077	−0.012	0.024	0.70036

### 4.1 Sensitivity analysis

A sensitivity analysis for the IVN fuzzy EDAS approach was carried out. Sensitivity analysis is used to evaluate the methodologies' accuracy and output. For the analysis, 35 criteria and 40 alternatives were examined systematically. A single parameter was varied at a time to determine the method's effectiveness, according to the findings of Canada [86]. The main criteria weights are systematically altered utilizing each linguistic term outlined in Table 3, while the alternative scores are observed. Table 12 elucidates the structure adopted for the sensitivity analysis. By utilizing this structure, the impact of the changes on the ranking of alternatives is assessed. Table 12 shows the graph of the sensitivity analysis for Criterion 1. Here, the numerical expressions corresponding to the linguistic values CL, VL, L, BA, A, AA, H, VH, and CH for 40 alternatives are shown in the graph. It can be seen that the values in the graph change in parallel with each other.

**Table 12:** Model developed for the sensitivity analysis.

Model		Sets with respect to criteria			
		Set 1	Set 2	…	Set m
**Test variables**	CL	Ranks	Ranks	…	Ranks
	VL	…	…	…	…
	L	…	…	…	…
	BA	…	…	…	…
	A	…	…	…	…
	AA	…	…	…	…
	H	…	…	…	…
	VH	…	…	…	…
	CH	Ranks	…	…	Ranks

**Fig. 4:**
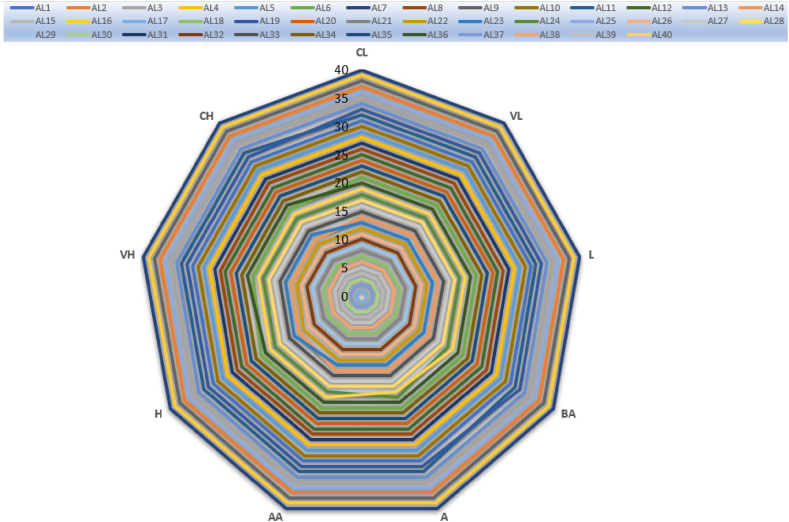
Result of the sensitivity analysis.

Fig. 4 displays the sensitivity analysis results for 40 different scenarios. Here, each linguistic variable is assigned to the criteria in turn. Then, new evaluation scores are obtained by repeating the other steps of the method. In other words, it is analyzed whether the rank numbers of the alternatives change according to the evaluation score. Fig. 4 graphically shows the result of the sensitivity analysis for SR1. Here, the verbal expressions corresponding to the linguistic values CL, VL, L, BA, A, AA, H, VH, and CH for each of the 40 alternatives are shown in the graph. It is seen that the values in the graph change parallel to each other. In other words, changes or deviations in the results were very small.

In Fig. 4, the values corresponding to all linguistic values of almost every alternative value remained the same in parallel. This indicates that the results and rankings are good. When the indicators changing depending on all alternatives and criteria are analyzed here, it is found that each criterion changes the alternatives. At the same time, all of the alternatives affect the evaluation score. When the graph was analyzed, the alternative with the best evaluation score remained the same for all tests. In other words, increasing or decreasing the criteria did not lead to a decrease or increase in the alternatives. When the sensitivity analysis results for IVN fuzzy EDAS are analyzed, it is seen that our proposed IVN fuzzy EDAS method gives accurate and consistent results in terms of decision-making.

As this study employs a novel method to address the inherent uncertainties associated with SRs in the automotive industry, a direct comparison with other established methods was not feasible. While classical MCDM models are effective in a variety of contexts, they often prove inadequate in addressing the inherent uncertainty and complexity of SRs. In light of these limitations, the present study was designed to validate the robustness and consistency of the IVN fuzzy EDAS approach through a comprehensive sensitivity analysis. This analysis is of particular importance in the context of this research, as it enables a systematic variation in the weights of the criteria, thereby facilitating an observation of the effects on the ranking of alternatives. By employing this approach, we were able to demonstrate that the proposed method produces reliable and stable outcomes even when the input parameters are altered. The results of the sensitivity analysis indicated that the rankings of the risk strategies remained consistent across different scenarios, which demonstrates the effectiveness of the method in managing SRs despite the lack of direct comparative benchmarks. This stability is essential for DMs in the automotive industry, as it confirms the method's capacity to provide consistent and dependable insights under varying conditions.

## 5 Conclusions and outlook

The concept of sustainability has gained significant traction in recent years, exerting a notable influence on various facets of Economic, Social, And Governance (ESG) considerations. The implementation of sustainability policies has been demonstrated to enhance organizational flexibility in decision-making and risk management. This is driven by a number of factors, including changing consumer demands, limited resources, supply chain risks, and climate change. This work introduced the IVN fuzzy EDAS method as a means of addressing SRs in the automotive sector. Conventional MCDM techniques frequently encounter difficulties in addressing the inherent uncertainty and complexity of SRs. The developed IVN Fuzzy EDAS method tackled these challenges by incorporating neutrosophic sets, thereby facilitating a more nuanced evaluation of SRs through the effective handling of indeterminate and inconsistent information. The model addressed five dimensions of sustainability: social, economic, environmental, managerial, and organizational. A comprehensive literature review was conducted, resulting in the identification of 35 SRs and the formulation of 40 risk prevention strategies. The most highly-ranked strategy placed an emphasis on the development of compliance procedures and programs designed to ensure adherence to existing regulations and principles, thereby guaranteeing sustainability through legal compliance and risk management. This approach highlighted the significance of compliance with legal and regulatory frameworks pertaining to risk management, business continuity, productivity, environmental risk management, human rights, economic efficiency, and operational and managerial sustainability. Examples of such measures included the adoption of the Universal Declaration of Human Rights, the implementation of compliance with legislation prohibiting child labor and discrimination, and adherence to international agreements such as the European Green Deal and the Paris Agreement. The second strategy entailed the commitment of top management to sustainable goals and the planning of business continuity, underscoring the necessity of establishing clear targets to ensure the sustained performance of sustainable practices. The third strategy emphasized effective planning and scheduling in logistics and supply chain processes, leveraging advanced technologies such as the Internet of Things, Smart Factories, and Cyber-Physical Systems. This procedure ensured efficient management practices, external relations, and information systems within logistics, fostering continuous improvement through agile working methods.

The evolution of the IVN Fuzzy EDAS method entailed the integration of Computing With Words (CWW) concept proposed by Orsato and Wells [85]. This concept deals with the handling of data expressed in common language and the uncertainty of information. Experts encountered difficulties in assigning intermediate linguistic terms due to the complexity of determining precise values. The majority of values tend to cluster around the mean, with a corresponding reduction in the number of options for extreme values. This phenomenon increases the degree of uncertainty as linguistic terms approach the mean. Accordingly, this research made a significant contribution to the existing literature on sustainability by addressing the problem from a number of different perspectives and introducing the IVN Fuzzy EDAS method to the context of SR management. The method's capacity to address ambiguity and uncertainty in risk factors provides a more precise and dependable instrument for DMs, offering a sophisticated approach to SR management that accommodates the industry's complexity. However, the IVN Fuzzy EDAS method's complexity and computational intensity present notable disadvantages. The implementation of IVN sets requires significant expertise and time, which may limit the method's widespread adoption without adequate resources. Nevertheless, the method's robustness and consistency, as demonstrated by the sensitivity analysis, highlight its potential as a valuable tool for enhancing sustainability in supply chains. The analysis confirmed that the rankings of alternatives remained consistent even when criteria weights were varied, underscoring the method's reliability.

All in all, this work offers a significant contribution to the field by introducing the IVN Fuzzy EDAS method to the domain of SR management in the automotive sector. The integration of organizational and managerial elements into the sustainability framework ensures a comprehensive strategy for addressing SRs, enhancing the applicability and actionability of the findings. Future studies might profitably undertake comparisons of this approach with other fuzzy sets, or indeed with other MCDM methods such as CODAS, TOPSIS, VIKOR, and AHP. Furthermore, the integration of diverse undertain decision-making models [[87], [88], [89], [90], [91], [92], [93], [94]] and risk assessment approaches [95–98] through hybrid approaches could enhance the precision and efficacy of SRs identification and management. In this regard, incorporating parameters such as probability, severity, and detectability of risks into these models could facilitate the development of comprehensive risk-based decision-making frameworks.

## Additional information

No additional information is available for this paper.

## Funding

The authors did not receive support from any organization for the submitted work.

## Human participants and/or animals (informed consent)

Informed consent was obtained from all individual participants included in the study.

## Ethical approval

Not applicable.

## Data availability statement

Google Scholar, Scopus, Science Direct, Web of Science, Emerald, Springer, and Elsevier databases were employed to collect the required data.

## CRediT authorship contribution statement

**Ecenur Alioğulları:** Writing – review & editing, Writing – original draft, Visualization, Software, Resources, Methodology, Investigation, Formal analysis, Data curation, Conceptualization. **Yusuf Sait Türkan:** Writing – review & editing, Visualization, Validation, Resources, Investigation, Formal analysis, Data curation, Conceptualization. **Emre Çakmak:** Writing – review & editing, Validation, Resources, Methodology, Formal analysis, Data curation, Conceptualization. **Erfan Babaee Tirkolaee:** Writing – review & editing, Validation, Investigation, Funding acquisition, Formal analysis, Data curation.

## Declaration of competing interest

The authors declare that they have no known competing financial interests or personal relationships that could have appeared to influence the work reported in this paper.
