# Supplementary material for: Evaluation of risk strategies for supply chain sustainability with interval-valued neutrosophic fuzzy EDAS
Source: Heliyon. 2024 Sep 27;10(19):e38607. doi: 10.1016/j.heliyon.2024.e38607 (PMC11471218; doi:10.1016/j.heliyon.2024.e38607)
Supplement: Multimedia component 1 [file mmc1.docx]

**Appendix A.** Supplementary tables

**Table A1.** Linguistic decision matrix for DM-1 (complete form of Table 6).

| **K/A** | **A1** | **A2** | **A3** | **A4** | **A5** | **A6** | **A7** | **A8** | **A9** | **A10** | **A11** | **A12** | **A13** | **A14** | **A15** | **A16** | **A17** | **A18** | **A19** | **A20** | **A21** | **A22** | **A23** | **A24** | **A25** | **A26** | **A27** | **A28** | **A29** | **A30** | **A31** | **A32** | **A33** | **A34** | **A35** | **A36** | **A37** | **A38** | **A39** | **A40** |
| --- | --- | --- | --- | --- | --- | --- | --- | --- | --- | --- | --- | --- | --- | --- | --- | --- | --- | --- | --- | --- | --- | --- | --- | --- | --- | --- | --- | --- | --- | --- | --- | --- | --- | --- | --- | --- | --- | --- | --- | --- |
| **K1** | CH | CH | CH | CH | VH | H | AA | H | AA | AA | AA | CL | CL | H | VH | CL | VH | AA | AA | AA | VH | VH | VH | VH | AA | A | BA | AA | H | VH | H | VH | VH | VH | H | H | VH | AA | AA | VH |
| **K2** | CH | CH | VH | CH | AA | CL | H | CH | CL | CH | VH | CL | CL | VH | CH | CL | CH | VH | VH | A | CH | VH | VH | VH | AA | A | A | A | H | VH | H | CH | VH | CH | CH | VH | CH | H | AA | CH |
| **K3** | VH | VH | VH | VH | CH | AA | VH | VH | A | VH | H | L | CL | L | BA | CL | H | AA | VL | CL | H | H | VH | A | A | A | A | A | H | H | H | H | VH | VH | AA | AA | H | AA | AA | VH |
| **K4** | AA | CL | A | A | A | CH | A | A | VH | A | A | A | CL | H | H | CL | VH | H | A | A | A | A | A | A | CL | A | A | CH | H | A | CL | A | A | A | A | A | AA | A | VH | H |
| **K5** | H | H | AA | VH | VH | AA | CH | CH | A | CH | H | CL | CL | CL | AA | CL | VH | AA | CL | A | H | AA | A | L | CL | A | L | H | L | A | VL | A | A | VL | BA | L | H | H | A | H |
| **K6** | VH | CH | CH | CH | H | A | VH | CH | BA | CH | VH | L | VL | CH | VH | CL | VH | H | VL | A | VH | VH | AA | H | AA | A | AA | H | VH | VH | L | VH | H | AA | H | H | VH | VH | H | VH |
| **K7** | L | CL | CL | AA | L | H | AA | AA | CH | VL | VL | CL | CL | CL | L | CL | VH | A | CL | L | H | L | VL | CL | BA | AA | VL | H | VL | BA | CL | L | CL | CL | L | VL | VH | AA | VL | H |
| **K8** | AA | AA | H | H | A | A | A | VH | VL | CH | VH | VL | VL | BA | BA | CL | AA | BA | CL | VL | AA | BA | L | A | L | A | A | BA | H | AA | L | H | A | H | A | A | H | A | AA | VH |
| **K9** | AA | A | A | VH | BA | A | L | VH | CL | H | CH | VL | VL | L | BA | CL | H | A | CL | VL | AA | BA | L | A | L | A | A | BA | H | AA | L | H | A | H | A | A | H | A | AA | H |
| **K10** | VL | VL | VL | VL | VL | H | VL | L | BA | CL | CL | CH | CH | VH | VH | AA | VH | VH | H | H | VH | BA | L | L | L | VH | A | H | A | A | A | A | A | A | A | A | H | H | BA | BA |
| **K11** | CL | CL | CL | CL | CL | VL | VL | VL | CL | VL | VL | CH | CH | H | VH | CH | CH | H | L | L | L | VL | VH | AA | BA | CH | BA | L | AA | A | VL | BA | L | L | VL | L | VH | AA | A | L |
| **K12** | VL | VL | VL | VL | CL | A | CL | BA | L | A | VL | H | H | CH | H | VH | VH | CH | AA | BA | H | BA | L | BA | L | VH | A | BA | A | AA | L | BA | A | A | L | A | VH | AA | A | L |
| **K13** | VL | VL | VL | VL | CL | BA | CL | VL | VL | VL | VL | VH | H | VH | H | CH | VH | AA | L | L | BA | L | BA | L | VL | CH | L | BA | L | AA | L | AA | BA | L | L | BA | H | BA | VH | L |
| **K14** | VL | CL | CL | VL | CL | AA | CL | VL | VL | VL | VL | H | H | VH | VH | VH | CH | H | VL | VL | L | L | L | VL | CL | CH | VL | BA | L | AA | L | L | VL | VL | L | A | H | A | VH | L |
| **K15** | BA | L | BA | BA | A | H | BA | A | AA | BA | L | VH | VH | H | H | VH | VH | H | VH | AA | AA | BA | AA | BA | L | VH | BA | H | AA | H | BA | A | A | L | L | A | H | H | H | A |
| **K16** | L | VL | VL | VL | VL | BA | CL | VL | CL | VL | VL | AA | L | BA | H | BA | H | L | CH | A | BA | L | AA | AA | VL | CH | AA | L | BA | A | BA | L | L | BA | L | L | H | A | H | BA |
| **K17** | BA | L | A | VL | L | L | A | BA | VL | VL | VL | L | L | BA | A | L | AA | BA | A | CH | BA | A | H | BA | A | A | AA | L | H | H | A | A | AA | H | AA | AA | VH | VH | VH | A |
| **K18** | L | L | L | BA | A | VH | VL | AA | AA | A | A | H | H | VH | H | A | AA | CH | AA | AA | CH | A | BA | A | VL | CH | BA | VH | A | A | BA | A | BA | L | L | A | H | A | BA | VL |
| **K19** | AA | BA | A | A | H | CH | BA | A | VH | BA | L | H | AA | AA | AA | H | VH | AA | H | VH | H | AA | AA | BA | A | CH | A | CH | AA | AA | BA | A | BA | L | A | L | H | AA | A | H |
| **K20** | AA | BA | A | AA | AA | AA | L | A | VL | A | A | BA | BA | BA | A | VL | VH | A | BA | AA | AA | H | CH | BA | CH | A | H | L | VH | H | AA | AA | A | AA | A | H | H | AA | AA | A |
| **K21** | VH | H | AA | A | BA | L | A | A | L | AA | AA | A | A | AA | A | A | AA | A | A | BA | A | H | CH | CH | AA | A | CH | A | VH | H | VH | AA | AA | H | A | A | VH | H | BA | AA |
| **K22** | L | VL | L | BA | L | L | VL | VL | VL | L | L | BA | A | AA | AA | CL | H | L | VL | AA | A | VH | H | H | CH | L | AA | VL | H | AA | A | AA | A | A | A | A | AA | A | AA | L |
| **K23** | VL | VL | VL | VL | VL | BA | VL | VL | CL | CL | CL | A | A | VH | H | AA | H | H | A | AA | AA | AA | AA | AA | AA | CH | A | A | VH | H | BA | AA | A | A | A | A | H | AA | VH | L |
| **K24** | H | BA | BA | BA | H | CH | BA | BA | H | BA | BA | L | L | BA | A | VL | AA | A | L | A | A | BA | BA | BA | L | L | AA | VH | H | H | BA | AA | AA | A | AA | AA | AA | AA | A | A |
| **K25** | AA | BA | BA | BA | BA | A | VL | BA | BA | BA | BA | A | A | A | A | BA | A | H | BA | A | A | AA | H | BA | BA | L | AA | BA | VH | CH | H | AA | AA | A | A | AA | VH | H | AA | BA |
| **K26** | L | L | L | BA | BA | A | VL | L | VL | L | L | VL | VL | BA | BA | VL | BA | BA | VL | BA | BA | A | A | VH | L | VL | BA | L | VH | H | CH | H | VH | H | H | VH | AA | AA | A | A |
| **K27** | BA | L | L | BA | BA | L | VL | L | VL | BA | BA | BA | VL | L | BA | VL | BA | BA | BA | A | BA | AA | A | AA | A | A | AA | BA | H | H | AA | CH | CH | AA | AA | H | AA | H | AA | A |
| **K28** | AA | AA | AA | H | H | H | A | AA | AA | H | AA | A | L | A | A | CL | AA | A | BA | A | A | A | AA | H | BA | A | A | A | VH | VH | VH | H | CH | VH | H | H | H | H | AA | AA |
| **K29** | AA | AA | AA | H | H | H | A | AA | AA | H | AA | A | L | A | A | CL | AA | A | BA | A | A | A | AA | H | BA | A | A | A | VH | VH | VH | H | CH | CH | H | H | H | H | AA | AA |
| **K30** | A | A | A | AA | A | H | L | A | BA | L | L | A | A | A | A | A | AA | A | A | H | A | A | VH | AA | VL | L | A | H | AA | H | AA | AA | A | A | CH | VH | AA | H | AA | A |
| **K31** | AA | AA | VH | VH | VH | H | H | VH | H | VH | VH | VH | H | VH | H | H | VH | VH | VH | H | VH | VH | VH | VH | AA | H | H | AA | VH | CH | H | H | H | H | VH | VH | H | CH | CH | H |
| **K32** | L | L | A | A | L | A | L | A | BA | A | A | VH | VH | VH | VH | H | VH | AA | AA | H | A | H | H | H | H | VH | H | H | AA | AA | H | AA | AA | A | A | A | CH | AA | VH | A |
| **K33** | AA | AA | H | H | H | H | AA | A | AA | AA | AA | A | A | H | H | A | H | AA | AA | VH | AA | AA | AA | AA | A | H | H | H | CH | CH | VH | CH | CH | H | VH | VH | CH | VH | H | AA |
| **K34** | AA | AA | AA | AA | AA | AA | AA | AA | VH | H | H | H | H | H | VH | H | CH | H | H | H | H | AA | H | VH | AA | VH | AA | H | VH | VH | VH | VH | VH | VH | VH | VH | VH | VH | CH | H |
| **K35** | VH | VH | VH | VH | H | H | H | VH | H | H | H | A | A | A | AA | A | H | A | H | AA | A | VH | AA | VH | AA | A | CH | AA | H | H | AA | AA | AA | AA | A | A | H | H | VH | CH |

**Table A2.** Values of *nsp_n_* and *nsn_n_* (complete form of Table 9).

| **Alternatives** | ***nsp_n_*** | | | | | | ***nsn_n_*** | | | | | |
| --- | --- | --- | --- | --- | --- | --- | --- | --- | --- | --- | --- | --- |
|  | TL | TU | IL | IU | FL | FU | TL | TU | IL | IU | FL | FU |
| **AL1** | -14.076 | 10.166 | 21.672 | 33.154 | -2.383 | 11.003 | 14.076 | -10.166 | 21.672 | 33.154 | 2.383 | -11.003 |
| **AL2** | -16.261 | 7.135 | 22.719 | 34.386 | -1.725 | 12.340 | 16.261 | -7.135 | 22.719 | 34.386 | 1.725 | -12.340 |
| **AL3** | -15.290 | 8.614 | 22.184 | 33.752 | -2.119 | 11.645 | 15.290 | -8.614 | 22.184 | 33.752 | 2.119 | -11.645 |
| **AL4** | -12.328 | 12.418 | 22.033 | 33.598 | -3.388 | 9.154 | 12.328 | -12.418 | 22.033 | 33.598 | 3.388 | -9.154 |
| **AL5** | -13.126 | 11.519 | 21.654 | 33.123 | -3.087 | 9.826 | 13.126 | -11.519 | 21.654 | 33.123 | 3.087 | -9.826 |
| **AL6** | -10.033 | 15.900 | 20.649 | 31.862 | -4.447 | 7.465 | 10.033 | -15.900 | 20.649 | 31.862 | 4.447 | -7.465 |
| **AL7** | -19.065 | 3.567 | 22.177 | 33.826 | -1.259 | 13.623 | 19.065 | -3.567 | 22.177 | 33.826 | 1.259 | -13.623 |
| **AL8** | -12.254 | 13.003 | 21.233 | 32.656 | -3.482 | 9.230 | 12.254 | -13.003 | 21.233 | 32.656 | 3.482 | -9.230 |
| **AL9** | -16.979 | 6.025 | 22.043 | 33.737 | -1.252 | 12.975 | 16.979 | -6.025 | 22.043 | 33.737 | 1.252 | -12.975 |
| **AL10** | -13.506 | 10.907 | 21.677 | 33.208 | -3.113 | 9.846 | 13.506 | -10.907 | 21.677 | 33.208 | 3.113 | -9.846 |
| **AL11** | -15.994 | 7.695 | 21.145 | 32.606 | -2.596 | 11.140 | 15.994 | -7.695 | 21.145 | 32.606 | 2.596 | -11.140 |
| **AL12** | -12.052 | 13.379 | 21.251 | 32.707 | -3.727 | 8.999 | 12.052 | -13.379 | 21.251 | 32.707 | 3.727 | -8.999 |
| **AL13** | -15.478 | 8.541 | 22.367 | 34.023 | -2.852 | 10.811 | 15.478 | -8.541 | 22.367 | 34.023 | 2.852 | -10.811 |
| **AL14** | -8.115 | 18.493 | 20.759 | 32.007 | -4.677 | 6.732 | 8.115 | -18.493 | 20.759 | 32.007 | 4.677 | -6.732 |
| **AL15** | -5.908 | 21.628 | 19.571 | 30.539 | -5.643 | 5.026 | 5.908 | -21.628 | 19.571 | 30.539 | 5.643 | -5.026 |
| **AL16** | -16.715 | 6.530 | 23.909 | 36.006 | -1.614 | 12.582 | 16.715 | -6.530 | 23.909 | 36.006 | 1.614 | -12.582 |
| **AL17** | 0.275 | 29.304 | 21.309 | 32.659 | -8.007 | 0.127 | -0.275 | -29.304 | 21.309 | 32.659 | 8.007 | -0.127 |
| **AL18** | -7.352 | 19.828 | 19.573 | 30.591 | -5.479 | 5.673 | 7.352 | -19.828 | 19.573 | 30.591 | 5.479 | -5.673 |
| **AL19** | -15.574 | 8.358 | 20.816 | 32.231 | -1.909 | 12.031 | 15.574 | -8.358 | 20.816 | 32.231 | 1.909 | -12.031 |
| **AL20** | -12.263 | 13.283 | 19.766 | 30.916 | -3.704 | 9.105 | 12.263 | -13.283 | 19.766 | 30.916 | 3.704 | -9.105 |
| **AL21** | -7.751 | 19.387 | 19.219 | 30.187 | -5.358 | 5.961 | 7.751 | -19.387 | 19.219 | 30.187 | 5.358 | -5.961 |
| **AL22** | -9.943 | 16.084 | 19.176 | 30.130 | -4.616 | 7.289 | 9.943 | -16.084 | 19.176 | 30.130 | 4.616 | -7.289 |
| **AL23** | -9.264 | 17.134 | 20.216 | 31.412 | -4.972 | 6.627 | 9.264 | -17.134 | 20.216 | 31.412 | 4.972 | -6.627 |
| **AL24** | -11.022 | 14.694 | 19.801 | 30.934 | -4.608 | 7.508 | 11.022 | -14.694 | 19.801 | 30.934 | 4.608 | -7.508 |
| **AL25** | -17.709 | 5.551 | 21.149 | 32.623 | -2.392 | 11.802 | 17.709 | -5.551 | 21.149 | 32.623 | 2.392 | -11.802 |
| **AL26** | -6.646 | 21.166 | 21.743 | 33.231 | -5.619 | 5.524 | 6.646 | -21.166 | 21.743 | 33.231 | 5.619 | -5.524 |
| **AL27** | -11.656 | 14.312 | 19.171 | 30.124 | -4.425 | 8.120 | 11.656 | -14.312 | 19.171 | 30.124 | 4.425 | -8.120 |
| **AL28** | -9.865 | 16.156 | 20.179 | 31.308 | -4.129 | 7.848 | 9.865 | -16.156 | 20.179 | 31.308 | 4.129 | -7.848 |
| **AL29** | -7.364 | 19.608 | 20.660 | 31.974 | -6.371 | 4.344 | 7.364 | -19.608 | 20.660 | 31.974 | 6.371 | -4.344 |
| **AL30** | -3.965 | 24.478 | 20.721 | 31.977 | -6.934 | 2.993 | 3.965 | -24.478 | 20.721 | 31.977 | 6.934 | -2.993 |
| **AL31** | -13.598 | 11.139 | 21.073 | 32.444 | -3.785 | 9.164 | 13.598 | -11.139 | 21.073 | 32.444 | 3.785 | -9.164 |
| **AL32** | -7.547 | 19.144 | 20.104 | 31.194 | -5.595 | 5.298 | 7.547 | -19.144 | 20.104 | 31.194 | 5.595 | -5.298 |
| **AL33** | -10.223 | 16.174 | 20.319 | 31.545 | -5.106 | 6.979 | 10.223 | -16.174 | 20.319 | 31.545 | 5.106 | -6.979 |
| **AL34** | -11.416 | 14.340 | 20.307 | 31.489 | -4.605 | 7.662 | 11.416 | -14.340 | 20.307 | 31.489 | 4.605 | -7.662 |
| **AL35** | -10.959 | 14.992 | 20.326 | 31.469 | -4.039 | 8.444 | 10.959 | -14.992 | 20.326 | 31.469 | 4.039 | -8.444 |
| **AL36** | -11.386 | 14.663 | 19.682 | 30.735 | -4.477 | 8.093 | 11.386 | -14.663 | 19.682 | 30.735 | 4.477 | -8.093 |
| **AL37** | -0.574 | 28.294 | 21.366 | 32.757 | -8.336 | -0.098 | 0.574 | -28.294 | 21.366 | 32.757 | 8.336 | 0.098 |
| **AL38** | -7.308 | 19.695 | 19.583 | 30.612 | -5.952 | 4.919 | 7.308 | -19.695 | 19.583 | 30.612 | 5.952 | -4.919 |
| **AL39** | -6.521 | 21.418 | 19.930 | 31.023 | -5.811 | 5.287 | 6.521 | -21.418 | 19.930 | 31.023 | 5.811 | -5.287 |
| **AL40** | -10.392 | 15.451 | 19.973 | 31.064 | -4.185 | 7.936 | 10.392 | -15.451 | 19.973 | 31.064 | 4.185 | -7.936 |

**Data availability statement**

Google Scholar, Scopus, Science Direct, Web of Science, Emerald, Springer, and Elsevier databases were employed to collect the required data.

**Additional information**

No additional information is available for this paper.

**CRediT authorship contribution statement**

**Ecenur Alioğulları:** Resources, Software, Visualization, Research, Writing - original draft, Formal analysis. **Yusuf Sait Türkan:** Formal analysis, Supervision, Verification, Writing- review & editing. **Emre Çakmak:** Formal analysis, Software, Writing -review & editing. **Erfan Babaee Tirkolaee:** Conceptualization, Formal analysis, Validation, Writing -review & editing.

**Declaration of competing interest**

The authors declare that they have no known competing financial interests or personal relationships that could have appeared to influence the work reported in this paper.

**Funding**

The authors did not receive support from any organization for the submitted work.

**Conflict of Interest**

Author Ecenur Aliogullari, Yusuf Sait Turkan, Emre Cakmak and Erfan Babaee Tirkolaee declare that he/she has no conflict of interest.

**Human Participants and/or Animals** (**Informed consent)**

Informed consent was obtained from all individual participants included in the study.

**Ethical approval:** Not applicable.

**Acknowledgment:** Not applicable.
